# The trophic distribution of biomass in ecosystems with co-occurring wildlife and livestock

**DOI:** 10.1038/s41598-025-85469-2

**Published:** 2025-01-09

**Authors:** James D. M. Speed, Anna Sobocinski, Anders L. Kolstad, John D. C. Linnell, Erling J. Solberg, Jenny Mattisson, Gunnar Austrheim

**Affiliations:** 1 Department of Natural History, NTNU University Museum, Norwegian University of Science and Technology, Trondheim, Norway; 2 Norwegian Institute for Nature Research (NINA), P.O. Box 5685, NO-7485 Trondheim, Norway; 3 Norwegian Institute for Nature Research (NINA), Vormstuguveien 40, 2624 Lillehammer, Norway; 4 Department of Forestry and Wildlife Management, University of Inland Norway, Koppang, Norway

**Keywords:** Carnivore, Herbivore, Management, Net primary productivity, Predation, Boreal ecology, Population dynamics

## Abstract

Trophic interactions regulate populations, but anthropogenic processes influence primary productivity and consumption by both herbivore and carnivore species. Trophic ecology studies often focus on natural systems such as protected areas, even though livestock globally comprise the majority of terrestrial vertebrate biomass. Here we explore spatial and temporal patterns in the distribution of biomass between plants, and large herbivores and carnivores (> 10 kg) in Norwegian rangelands, including both wildlife and livestock. We find high spatial variation in the relationship between plant and herbivore biomass, with both positive and negative divergence in observed biomass from expectations based on primary productivity. Meanwhile, despite recent partial recoveries in carnivore densities across Norway, carnivore biomass is still lower than expected based on herbivore biomass, even if livestock are excluded from the estimation. Our study highlights how temporal trends in both herbivores and carnivores reflect policy development. The role of livestock husbandry and wildlife management is thus key in determining realised biomass distributions in anthropogenically influenced ecosystems.

**Supplementary Information:**

The online version contains supplementary material available at 10.1038/s41598-025-85469-2.

## Introduction

Energy flows through ecological communities from primary producers to top-level consumers. This leads to pyramidal patterns of biomass across trophic levels with remarkably consistent biomass scaling between prey and predators^1^ and between herbivores and vegetation^2,3^. At the same time, consumers can influence the biomass of lower trophic levels through top-down cascades^4,5^. Bottom-up energetic flows and top-down trophic cascades may vary in importance between ecosystems and over time and space^6,7^. Trophic interactions determine the structure of communities, ecosystems and even biomes, and play a huge role in ecosystem functioning with impacts on global climate^8,9^.

Anthropogenic activity alters trophic networks through a range of impacts, including climate change, direct exploitation, agriculture, forestry, land-use change, pollution and the spread of non-native species^10^. Global environmental change may affect bottom-up energetics. For example, global warming or nitrogen deposition may increase primary productivity in temperature-limited environments^11,12^, . Changes in productivity can also cause shifts in vegetation composition, such as shrub expansion in the tundra^13^, which can alter consumer dynamics^14^. Land-use changes and agricultural policies influence the prevalence of natural ecosystem, wildlife and livestock. Anthropogenic changes can also affect cascading properties of trophic interactions. Wildlife management influences the degree of human harvest, or supplementary feeding, of game species. This can be through the selective removal of top predators from ecosystems, which can lead to increasing densities of herbivores^8,15^ and meso-predator release^16^.

Global biomass distributions have been changed to a state where the vast majority of non-human mammal biomass today is comprised of a few species of livestock^17,18^. Recently, in some regions, notably Europe, a combination of social, economic, legal and political drivers have driven rural land abandonment, a decrease in livestock densities, and increases in wild herbivores^19,20^ and carnivores^21^.

Empirical studies investigating biomass distributions within ecological communities have generally focused on systems that retain natural communities, such as national parks and restored or rewilded systems^1,2^. However, in many (if not most) parts of the world, free-ranging livestock and wild species overlap in distribution^22^. Wild herbivores and livestock are under different management regimes within the same areas but have a common impact on primary production. Thus, it is important to understand how wildlife and livestock fit into trophic biomass distributions for a more integrated nature and agricultural management.

In this study we investigate how biomass distribution varies across time and space between trophic levels. We focus on the outfields, or rangelands of Norway (termed *utmark* in Norway); the unimproved and unenclosed land mostly comprising alpine and arctic tundra and forests. The study concentrates on large vertebrate consumers (> 10 kg), including livestock and wildlife, as well as vegetation. Norway is a highly suitable study system having good historical data on wildlife and livestock populations and a long history of wildlife and livestock dynamics across ecosystems^20,23^. In Norwegian forests, herbivores have undergone a shift from livestock dominance in the 1950s to parts of the country now having predominantly wild herbivore populations, while alpine regions remain largely dominated by livestock^20^. Large carnivore populations have shown some recent recovery in Europe^24–26^ In Norway, large carnivore populations (wolf *Canis lupus*, brown bear *Ursus arctous*, wolverine *Gulo gulo* and lynx *Lynx lynx*) have staged a partial recovery since the cessation of state bounties in the 1970’s and 1980’s, but densities remain very low due to the widespread use of lethal means to limit their populations to politically determined numbers and distributions to specific zones^25,27,28^, later referred to as carnivore management zones.

This analysis has the following objectives: First, to quantify the biomass of large herbivores and carnivores in Norway as far back in time as possible, second, to compare observed biomass of consumer guilds with the biomass at lower trophic levels, based on published trophic biomass scaling relationships^1,2^ and third to answer the following questions: 1. Are herbivore and carnivore biomass as expected given net primary productivity (NPP), and if not, where do deviations exist? 2. Is realized carnivore biomass in line with expectations based on wild herbivore populations (i.e. carnivore populations are at a level appropriate for wild prey)? We predict (1) that herbivore biomass is on average in line with expectations from NPP, albeit with regions with higher and lower biomass than expected (i.e. over- or under-grazing), and (2) that carnivore biomass is lower than expectations from realized (and expected) herbivore biomass, but with lower deviation in carnivore management zones.

## Results

### Biomass within trophic levels

**Fig. 1:**
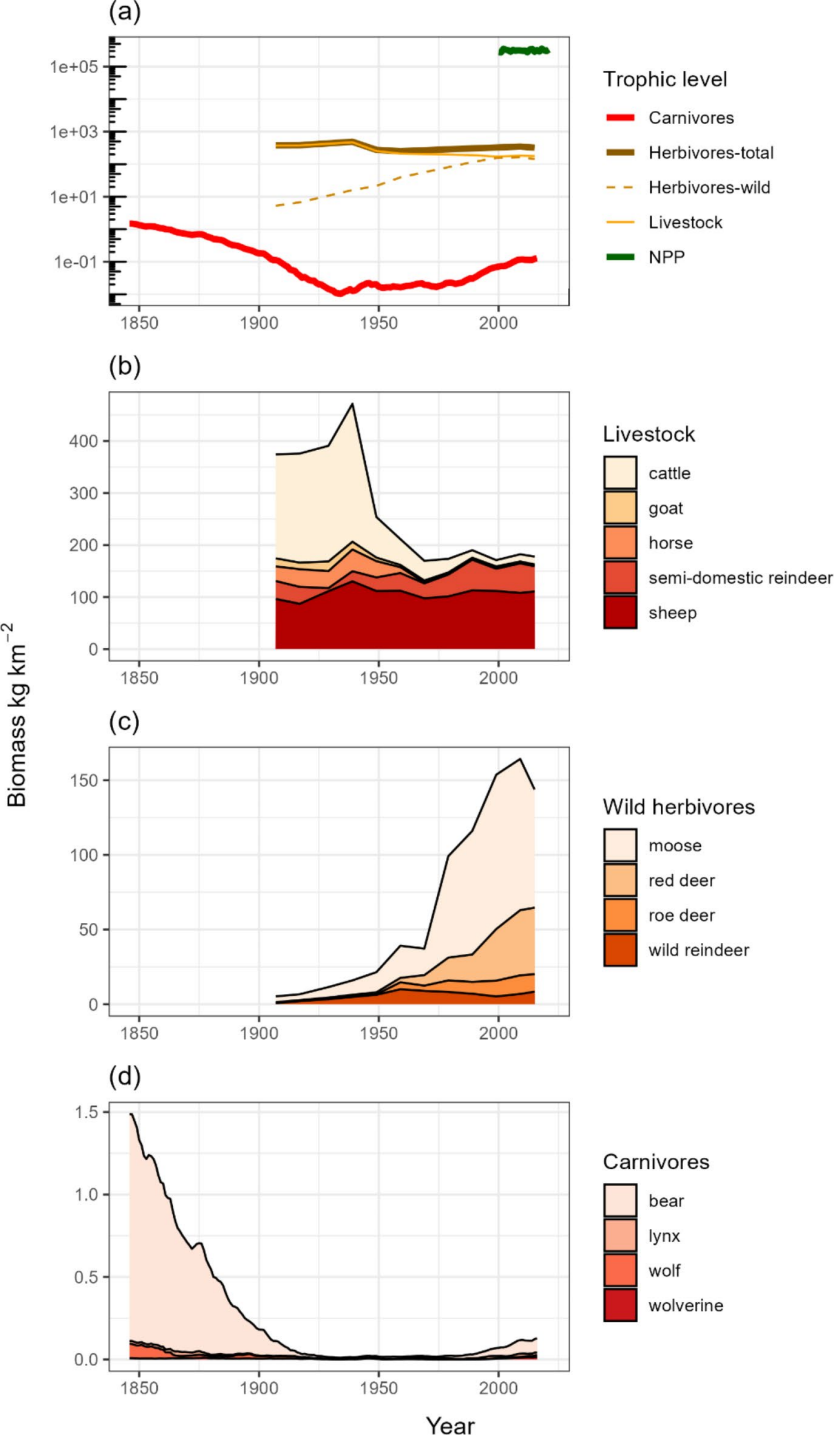
Biomass trends in trophic levels across Norway. (**a**) Shows the average biomass density per trophic levels vegetation (as NPP, 2000–2021, see also Figure S1), large herbivores (> 10 kg 1907–2015) and large carnivores (> 10 kg, 1846–2015); the contributions of livestock and wild herbivores to total herbivore biomass are indicated. (**b**) and (**c**) show the species contributions to biomass of livestock and large wild herbivores. Part (**d**) shows the contribution of carnivore species to total carnivore biomass. Note the different extents of the data series on the x axis, and that the y axis in part (a) is log_10_ transformed, while the others are linear.

**Fig. 2:**
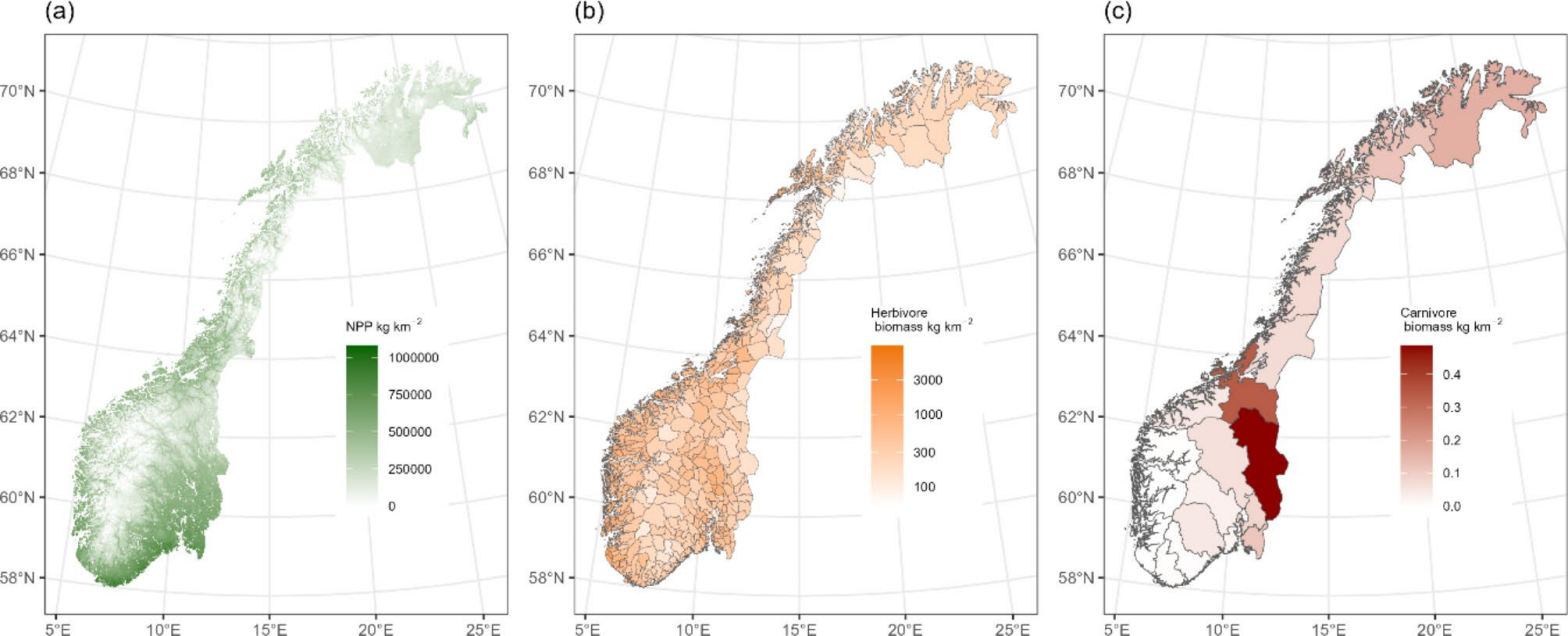
Spatial distribution of biomass across three trophic levels in Norway in 2015. NPP is quantified and visualized in 500 m cells (**a**), herbivore biomass within municipalities (**b**) and carnivores at county level (**c**). Maps created in R^58^, using the packages sf^59^ and terra^60^.

Net primary productivity across Norway varied from a mean of 272 000 kg C km^− 2^ in year 2001, to 357 000 kg C km^− 2^ in year 2018 (Fig. 1a, Figure S1). NPP was highest in the lowlands in the southeast of the country and along the coast, peaking at over 1 000 000 kg C km^− 2^, and lowest in mountain regions and in the north (Fig. 2a; Figure S2).

Herbivore biomass at the national scale was at its highest in 1939 at 488 kg km^− 2^ and was lowest in 1959 at 250 kg km^− 2^ (Fig. 1a). There was a high turnover of species within the herbivore guild. In 1939 livestock (mostly cattle) comprised 97% of herbivore biomass across Norway while in 2009 livestock comprised 53% of the total herbivore biomass (mostly sheep *Ovis aries*, Fig. 1a and b). This turnover mainly comprised a replacement of cattle with the wild cervid species moose (*Alces alces*), red deer (*Cervus elaphus*) and roe deer (*Capreolus capreolus*; Fig. 1b and c). In 2015, herbivore biomass was highest in municipalities along the west coast of Norway and in northern Oppland county (Fig. 2b, see Figure S3 for map of counties). Herbivore biomass density maps of all species across the study period are shown in Figure S4.

Carnivore biomass was highest at the start of the period of data availability, in 1847 at 1.32 kg km^− 2^ (Fig. 1d). The minimum carnivore biomass of 0.009 kg km^− 2^ was reached in 1934 before rising again to around 0.12 kg km^− 2^ in recent years. In 2015 carnivore densities were highest in eastern and northern counties of Norway (Fig. 2c). Carnivore biomass density maps of all species across Norway between 1846 and 2015 are shown in Figure S5.

### Biomass between trophic levels

**Fig. 3:**
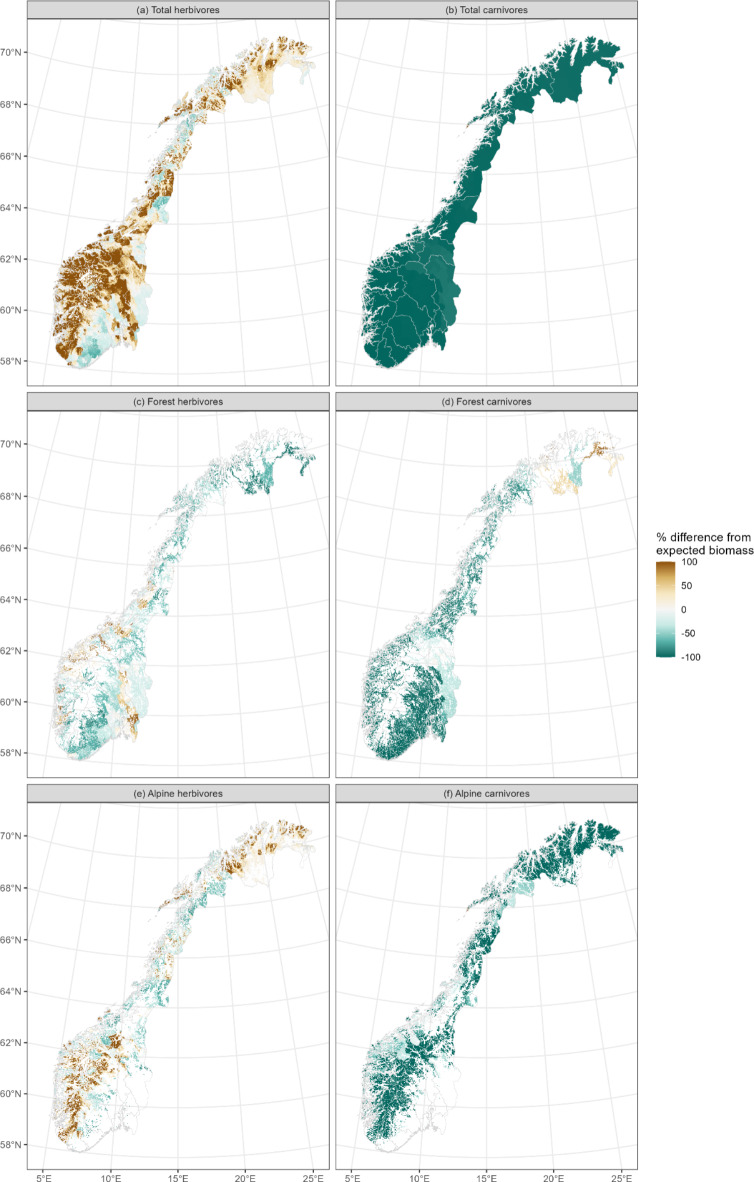
Deviation between observed and expected biomass of herbivores (left, (** a**,**c**,**e**)) and carnivores (right, (**b**,**d**,**f**)) across all species and ecosystems (top) and specific to forest species and ecosystem (middle) and alpine species and ecosystems (bottom). Data are from the year 2015. Maps created in R^58^, using the package sf^59^.

In 2015, the biomass density of all herbivores was higher than expected based on NPP in most of Western and Mid-Norway as well as in Northern Norway (Fig. 3a). At the same time, herbivore biomass density was lower than expected in South Norway, the coast of Nordland and the mountains of northern Trøndelag. Concurrently, carnivore densities were lower than expected based on observed herbivore densities across the whole of the country, except for Hedmark and southern Trøndelag (Fig. 3b).

Within forest ecosystems (and forest consumer species) herbivore biomass was generally in line with or lower than expected across much of the country in 2015. The southern counties of Telemark and Aust- and Vest-Agder had the largest negative deviation from expected herbivore biomass, along with Finnmark in north Norway. In contrast, there was higher forest herbivore biomass than expected based on NPP in Akershus, Østfold and parts of Oppland (Fig. 3c). Within alpine ecosystems, there were higher biomass densities than expected in the mountains in the south, as well as in Finnmark. In Trøndelag and Nordland, alpine herbivore biomass densities were generally lower than expected based on NPP (Fig. 3e). In both forest (Fig. 3d) and alpine (Fig. 3f) ecosystems, carnivore biomass densities in relation to herbivore biomass densities were similar to the overall pattern, with lower carnivore densities than expected in most of the country, with the exception of Hedmark which had forest carnivores in line with expectations from forest herbivores (Fig. 3d) and Finnmark, where forest carnivores were higher than expected given the densities of forest herbivores (Fig. 3d).

**Fig. 4:**
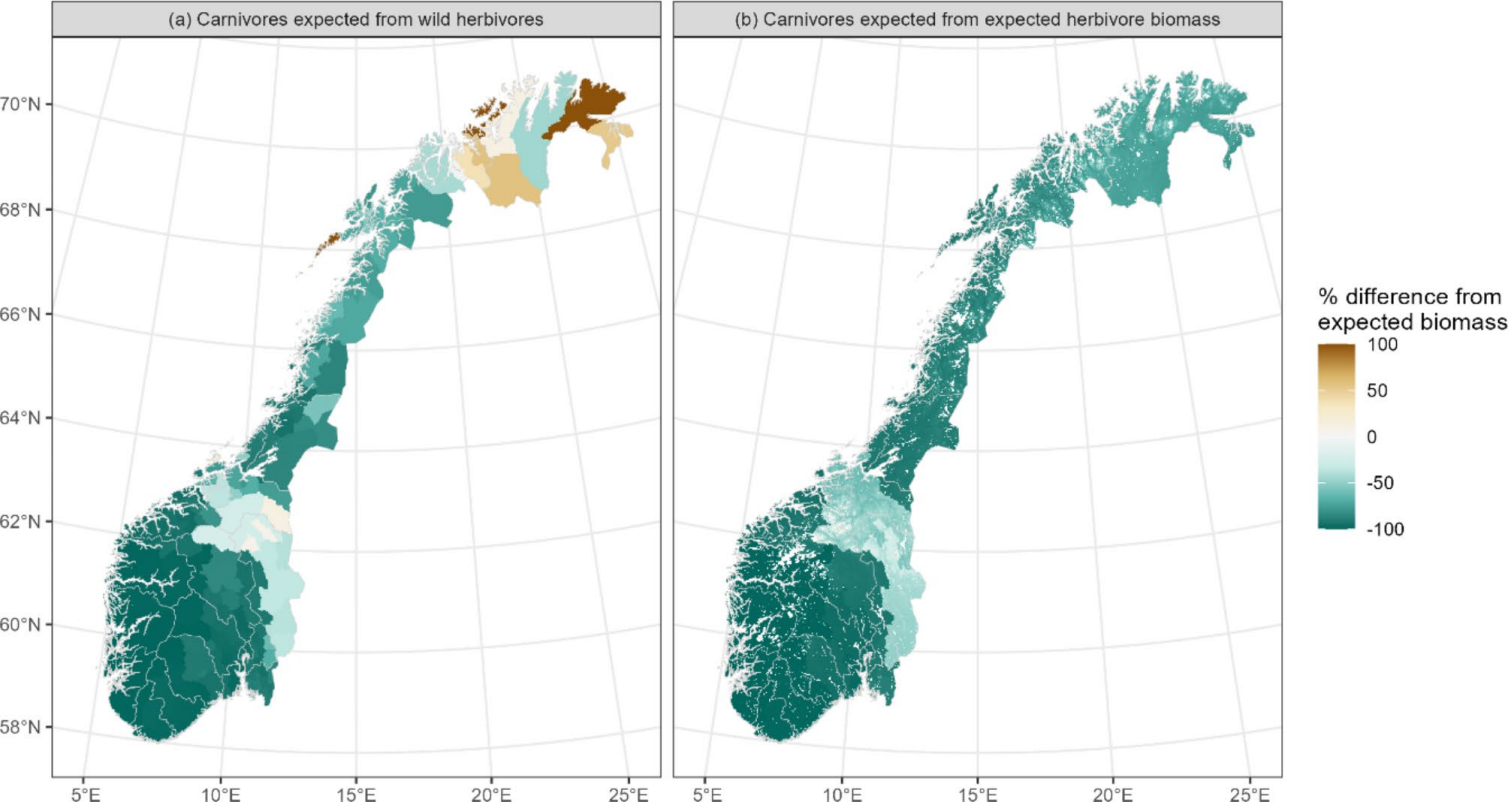
Deviations between observed and expected carnivore biomass based on wild herbivore populations (left, (**a**)) and expected herbivore biomass (i.e. from NPP; right, (**b**)). Maps created in R^58^, using the package sf^59^.

Carnivore biomass in 2015 remained below expectations if expected carnivore biomass is estimated based on only wild herbivore biomass (i.e. excluding livestock) across most of the country (Fig. 4a). There are exceptions in Hedmark and southern Trøndelag where carnivore biomass is in line with wild herbivore biomass and Finnmark where carnivore biomass is higher than expected (Fig. 4a). Carnivore biomass is also lower than expected based on expected-herbivore-biomass (Fig. 4b), again with the exception in Hedmark and southern Trøndelag where carnivore biomass is in line with expectations.

**Fig. 5:**
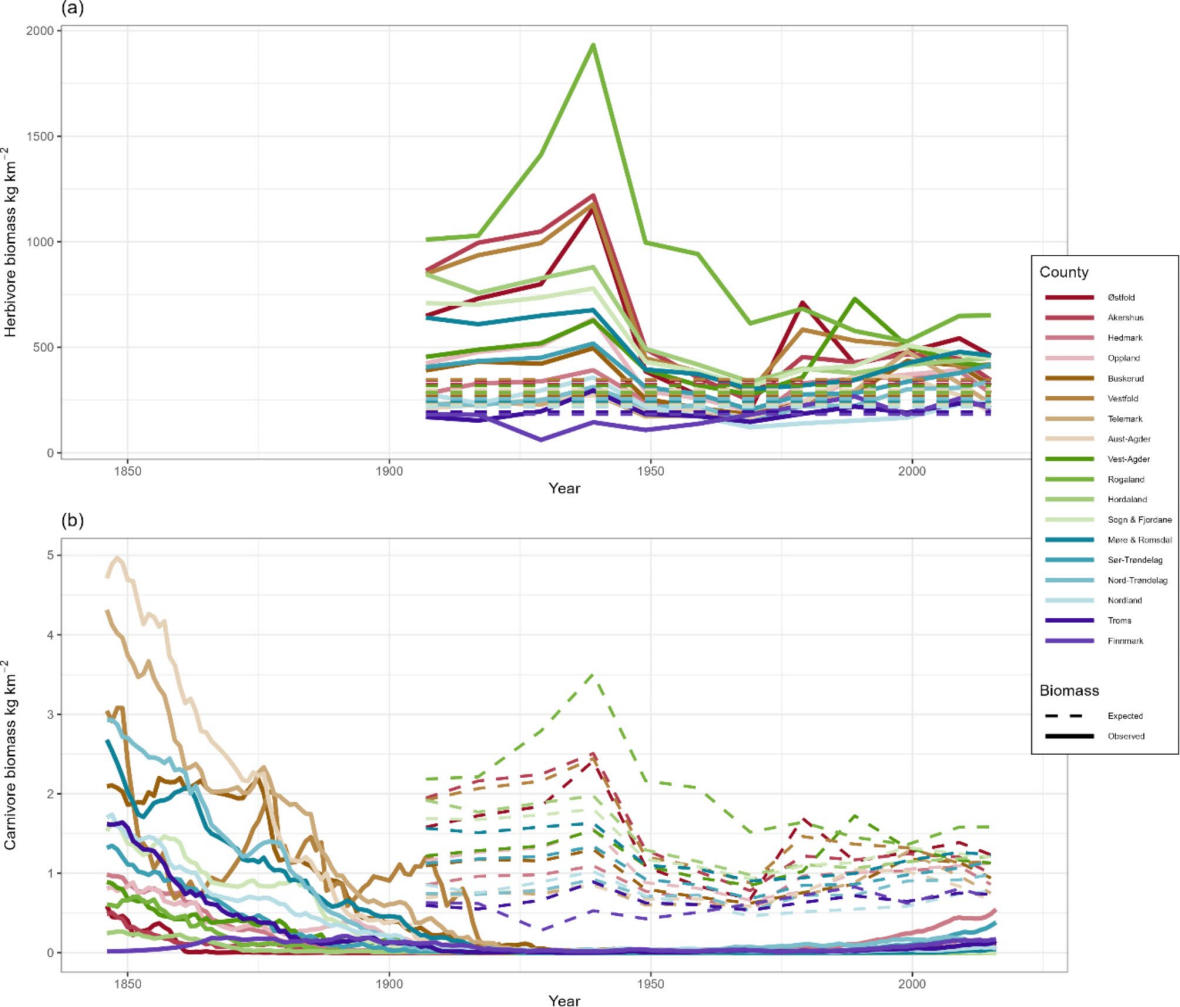
Temporal trends in herbivore (top, (**a**)) and carnivore (bottom, (**b**)) biomass across Norway’s 18 counties (excluding Oslo). Expected biomasses are shown as dashed lines. For expected herbivore biomass this is based on the average NPP across 2000–2021. For carnivores, the expected biomass is calculated based on herbivore biomass (1907–2015).

Data underlying the three trophic levels was available for different time periods, limiting analysis of temporal trends in biomass deviations from expectations. However, prior to the 1950s, some counties in the south and west of Norway, had far higher herbivore biomass than expectations based on NPP estimated during 2000–2015 (Fig. 5a). In contrast, in northern counties such as Troms and Finnmark, herbivore biomass (mostly semi-domestic reindeer; Figure S4) remained in line with expectations based on NPP (estimated only between 2000 and 2015). Carnivore biomass was lower than expected based on herbivore biomass for all counties for the whole period in which herbivore data was available (Fig. 5b). However, prior to the 1920s, carnivore biomass was in line with what would be expected for herbivore biomass in the period 1929–2015, and in some counties, including Telemark and Aust-Agder, higher than expectations.

## Discussion

In this study we present biomass estimates of carnivores and herbivores across space and time. We show that the biomass of herbivores and carnivores fluctuated over time. Herbivore biomass varied greatly in relation to expectations based on net primary productivity, while throughout the 20th and 21st centuries, carnivore biomass was lower than expected based on both observed and expected herbivore biomass. This study provides an integrated assessment of trophic structure at a national spatial scale and centurial temporal scale. Trophic structure is an important component of ecosystem function. Our study builds on previous studies^1,2^ by highlighting the need to account for both livestock and wildlife, since in our study region, livestock accounted for over half of the total herbivore biomass during the 20th century. The co-occurrence of livestock and wildlife is not specific to Norway, but is common throughout the world, and not surprising given the predominance of livestock in animal biomass^17^. In many rangelands livestock comprise the majority of herbivore biomass, for example 86% across Iceland^29^. However, many ecological studies still attempt to focus solely on “natural” ecosystems and dynamics^30^ ignoring the huge influence of livestock on ecological processes.

Herbivores shifted from livestock dominance in Norway during the early to mid-20th century, to approximately equal densities of wildlife and livestock at the end of the 20th century and into the 21st century; this expands the timeframe of the dynamics earlier reported^20,31^. Our analyses suggest that parts of the mountain regions have a greater herbivore biomass than expected given the vegetation productivity, filling one of many perspectives on the concept of overgrazing^32^. The high herbivore densities seen in the Norwegian mountains have been maintained for many decades in these regions (Fig. 5) with impacts on ecosystem functioning^33^. The maintenance of high herbivore densities in Norwegian mountains today likely has strong social and economic basis, calling for integrating historical context with ecology in understanding interactions between ecosystems and consumers^33,34^.

The distribution of biomass between trophic levels has been used as an indicator of ecosystem condition^35^ and both herbivores and carnivores are important contributors to ecosystem services^36^. Our results show that carnivore densities are lower than expectations based on herbivore biomass across Norway today, and have been since at least the 1920s, except for the eastern and northern parts of the country. Carnivores declined in the late 19th century due to intense state-sponsored persecution, were at a nadir throughout most of the mid-20th century, when the wolf was functionally extinct, bears were reduced to non-breeding occurrences and both wolverines and lynx were reduced to small remnant populations. Decades of changes to legislation has led to the return of breeding populations of all species during the late 20th century, largely within limited carnivore management zones^26^. Wolf and bear numbers remain low and are limited to counties with carnivore management zones, mostly along Norway’s eastern border. Lynx and wolverines are more widespread. However, lethal control and hunter harvest are used to maintain numbers and distributions within strict politically determined limits and zones, to reduce depredation of livestock. Carnivore management is a highly emotive and controversial issue in Norway^37^. Livestock losses to predation are substantial^38–40^ and livestock production practices have to adapt to carnivore presence^41^. The lower than expected carnivore biomass, even when considering only wild herbivores, suggests that Norwegian ecosystems have a reduced integrity and resilience compared to a natural state^35^. Impacts of deviations in trophic functioning on ecosystems can cascade across guilds of carnivores, herbivores and vegetation, with impacts also on biodiversity, soils, disease spread and on biogeochemical processes^36^.

In this study, analyses were performed across all ecosystems and species, but separately for the main ecosystems: forest and alpine. The trophic levels include species that predominantly use different ecosystems for example moose (forest) and reindeer (alpine). When analyses are separated by ecosystem type (across all three trophic levels), we arrive at the conclusion that forest herbivore biomass is lower than expected in Finnmark. Moose, red deer and roe deer have historically been absent from the far north of Norway, but now are partly colonizing the region. However, semi-domestic reindeer use forest habitats in Norway, especially in winter^42^, thus the herbivore trophic level is likely underestimated for the forest ecosystem in Finnmark. This exemplifies the habitat flexibility of some species implying that the habitat-specific results should be interpreted with caution.

The scale at which biomass of each trophic level was assessed varied, from 0.25 km^2^ for primary productivity, through municipalities for herbivores (median rangeland area of 418 km^2^) to counties (median rangeland area of 14 000 km^2^) for carnivores. This assumes that consumer habitat use is constant across the area assessed. This is unlikely to be the case, as both herbivores and carnivores are selective in their habitat use^43–45^. In addition, there are likely unaccounted errors in backcasting carnivore populations from hunting statistics, such as vehicle killings and illegal hunting. Our modelled estimates were reasonably similar to monitoring-based estimates of carnivore populations for bear and wolf. The models underestimated lynx and wolverine populations during a period of rapid increase (Figure S6) and may also overestimate populations in periods of population decrease.

We used published global biomass scaling models to estimated expected biomass at herbivore and carnivore trophic levels. The herbivore model used had a low coefficient of determination (0.04; Ref.^2^) with few sites in boreal or tundra ecosystems, which is a potential source of error. We also applied models developed predominantly in natural ecosystems (mostly national parks and protected areas), to a system with co-occurring livestock and wildlife. While we did standardise biomass estimates by the fraction of the year that livestock are in the rangelands, a caveat remains; that livestock populations in Norwegian rangelands, as in other northern systems, are subsidised through the winter season by supplementary feeding^46^. In addition, wild herbivore species can be subsidised through foraging on fertilized agricultural crops^47^. The absence of many of the livestock species (excepting semi-domestic reindeer) from ecosystems during the winter also has implications for the relationship between carnivore biomass and herbivore biomass. However, carnivore biomass was below expectations based on only wild herbivore biomass throughout most of the country (Fig. 4a). Similarly, bears hibernate during winter and have an omnivorous diet. Since bears contribute a high proportion of carnivore biomass, this increases the conservatism of our finding of lower carnivore biomass than expected from herbivore biomass. Both herbivore and carnivore guilds analysed here were limited to large species with an average biomass of over 10 kg. Herbivorous rodents, lagomorphs and avian and invertebrate herbivores fell outside this definition, yet all can influence vegetation dynamics in northern ecosystems. Thus, our analyses do not capture total vegetation-herbivore dynamics, only the species managed as game and livestock. Among the carnivores, golden eagles (*Aquila chrysaetos*) and red foxes (*Vulpes vulpes*) may rarely kill young of the smaller herbivore species considered, yet we assume that this seasonally restricted predation is limited enough that our study retains relevance for dynamics of managed species.

Livestock management is driven by economic and regulatory mechanisms in the region^33,42^. Populations of wildlife are also heavily influenced by human management, with sex and age specific hunting quotas used as management tools to influence population growth rates^48,49^. Thus, while our study focusses on biomass ratios between plants, herbivores and carnivores, all of these trophic levels are under strong human influence. Norway has been identified as having the greatest potential for rewilding in Europe^50^, however, given that herbivore biomass is already high relative to productivity, and carnivore management is heavily dictated by livestock concerns, the likelihood of enacting such a shift is very low.

Our approach identifies regions of Norway which have relatively higher or lower consumption across trophic levels, with relevance for livestock, wildlife and ecosystem management. The scarcity of carnivores in Norwegian ecosystems in relation to herbivore biomass likely has implications for ecosystem structure and functioning, biodiversity and ecosystem services^8^. The impact of reintroduced carnivores on carbon storage in North America has been estimated to be negative in grasslands but positive in forest ecosystems^51^. In Norway, high densities of forest herbivores (Fig. 2), reduce aboveground carbon storage by supressing tree biomass during secondary succession; this has potential feedbacks to climate^52^ as well as potential interactions with forest management^53^. Higher carnivore densities would be expected to limit forest herbivore populations leading to increased tree growth and carbon storage, but due to increased albedo under browsed conditions, especially during the snow-covered winter, the net climate effect of increasing carnivore densities in forests may be neutral^54^.

In conclusion, our study shows that across large parts of Norwegian rangelands, herbivore biomass is high in relation to primary productivity and large carnivore biomass is low in relation to primary productivity. However, there is a high degree of spatial variation in these patterns. In ecosystems with co-occurring wildlife and livestock, dynamics are influenced both by natural trophic dynamics, acting in concert with management of ecosystems and single species. This calls for integrated management involving forestry, agricultural and wildlife sector stakeholders.

## Methods

### Biomass of trophic levels

Plant, herbivore and carnivore biomass were each quantified across Norwegian rangelands (uncultivated land mainly comprising forests and tundra above the latitudinal and elevational treelines). Herbivore and carnivore biomasses were estimated on the basis of hunting and livestock statistics and records. For herbivores these data were available at the municipality level and for carnivores at the county level. Administrative boundary changes have occurred (mostly merging of units). For both municipality and county data, boundaries in place prior to 2017 were used and are discussed in the text (Figure S3).

Plant biomass was quantified as net annual primary productivity (NPP). While productivity is a rate (biomass increment over time), this was deemed most appropriate since annual productivity is assumed to be more available to herbivores than standing plant biomass, of which a large pool is held in woody biomass. Furthermore, NPP has previously been used to predict global patterns in herbivore biomass^2,3^ and is thus required to estimate expected herbivore biomass. Annual NPP was downloaded from MODIS at 500 m spatial resolution, spanning the years 2000–2021^55^. The global dataset was cropped and masked to the country of Norway, and NPP converted to kg C km^− 2^ yr^− 1^.

Herbivore biomass was estimated for the period from 1907 to 2015 for species over 10 kg. The approach extended the datasets presented by^20,31^, which were previously limited to 1949–2015. Agricultural statistics reports and reindeer herding reports were used to quantify livestock population biomass, while wild herbivore population biomass was quantified on the basis of population models parameterised by hunter-observations and hunting statistics. In contrast to the aforementioned studies, herbivore biomass was estimated as raw biomass densities rather than metabolic biomass densities. Biomass densities were quantified for livestock species (cattle *Bos taurus*, sheep *Ovis aries*, goats *Capra hircus*, horses *Equus ferus caballus* and semi-domestic reindeer *Rangifer tarandus*), and wildlife species; (moose *Alces alces*, roe deer *Capreolus capreolus*, red deer *Cervus elaphus* and wild reindeer *Rangifer tarandus*). Note that reindeer in Norway are comprised of both wild populations and semi-domestic populations (both are *Rangifer tarandus* ssp. *tarandus*) and due to their separate status and management are treated separately here. A small population (around 200 individuals) of muskox (*Ovibos moschatus*) exists in the Dovrefjell mountains in Central Norway which management authorities consider a non-native species^20^, and was subsequently omitted from this study. Prior to 2015, individual wild boar (*Sus scrofa*) were only occasionally observed in south-eastern and central Norway and due to extremely low and sporadic numbers at that time, are also omitted from this study.

Herbivore biomass densities were estimated for Norwegian municipalities at a decadal resolution (Table S1), using the pre-2017 administrative boundaries, and summing data where required to reflected merged municipalities. Livestock densities were standardised by the fraction of the year that each species spends in the rangelands (i.e. the population biomass for a species ranging for 3 months of the year would be standardised to 25%). Austrheim et al.^31^ provide further methodological details on the estimation of herbivore biomass densities.

Carnivore biomass was estimated using a backcasting population dynamic model based on the approach used for Finnish carnivores by Mykrä and Pohja-Mykrä^56^. All species of large carnivores (over 10 kg) in Norway were included: the grey wolf *Canis lupus*, wolverine *Gulo gulo*, brown bear *Ursus arctos* and Eurasian lynx *Lynx lynx.* Carnivore biomass estimates were based on hunting statistics at the county level (using county boundaries in existence prior to 2018) and covered the period 1847–2016. While this method using hunting statistics is likely to be error prone and makes the assumption that hunted individuals are accurately reported (the early bounty system and modern-day wildlife management practices facilitated fairly accurate and complete recording), it remains the best and only approach to quantify past population dynamics at this resolution^56^. Appendix A gives full methodological details regarding modelling carnivore biomass densities. Backcasted carnivore populations are shown in Figure S6. Carnivore data was estimated at an annual resolution, but is presented at a decadal resolution, matching that of herbivores (Table S1).

### Expected consumer biomass

The expected herbivore biomass (HB_exp_) based on plant productivity was calculated using the global relationship published by Fløjgaard et al.^2^. This global relationship (Expected herbivore biomass = 0.643 × NPP^0.47^) was not as strong as relationships derived from African ecosystems^2^, however it was deemed most appropriate due to the present day, and historical, lack of mega-herbivores in Norway. The global relationship was selected rather than the European and North American relationships as the range of productivity values covered by the global model had a closer match with NPP in Norway (the European relationship had no datapoints, and the North American relationship had only five data points below the mean NPP of Norway).

$$\:{HBexp}_{i}={NPP}_{i}^{0.47}\times\:0.643$$

[Eq1] from Fløjgaard et al.^2^, where HBexp_i_ is expected herbivore biomass in year *i*, and NPP is the NPP in year *i.*

Expected carnivore biomass (CB_exp_) based on herbivore biomass was based on the scaling relationships published by Hatton et al.^1^. We used the relationship Carnivore biomass expected = 0.094 × Herbivore biomass^0.73^ from African communities noting that the scaling exponent was near identical in wolf-prey systems [0.72, Ref. 1].

$$\:{CBexp}_{i}={HB}_{i}^{0.73}\times\:0.094$$

[Eq2] from Hatton et al.^1^, where CBexp_i_ is the expected carnivore biomass in year *i* and HB is the herbivore biomass in year *i*.

Deviations between observed herbivore biomass and expected herbivore biomass were calculated for 2000 (using observed herbivore biomass from 1999 with expected herbivore biomass in 2000), 2009 and 2015. Deviations between observed carnivore biomass and expected carnivore biomass were calculated for the common period of 1907–2015. Older carnivore biomass data are also presented here to visualize longer-term dynamics.

Biomass deviations were calculated across all species and limited to forest and alpine ecosystems. The ecosystem-specific estimates were based on NPP cells assigned to each major land-cover class using the AR50 land-cover map^57^ and the classes *skog* (forest) and *snaumark* (open vegetation). The open category includes land above the elevational and latitudinal treeline as well as less extensive lowland heaths. Consumer species were assigned according to their predominant habitat as follows: Moose, red deer, roe deer and cattle were classed as forest herbivores and wolf, bear and lynx as forest carnivores. Wild reindeer, semi-domestic reindeer, sheep and goats were classed as alpine herbivores and the wolverine as an alpine carnivore (horses were not included in either ecosystem). We note that most of these species use multiple habitats, however, we do not have the data to assign species’ proportional habitat use accounting for spatial and temporal variation. Finally, to address the question of whether carnivore biomass is in line with wildlife biomass, we estimated expected carnivore biomass based on only observed wild herbivore biomass (Eq. 3), and to address the question of whether carnivore biomass is in line with NPP, we estimated expected carnivore biomass as a function of expected herbivore biomass (Eq. 4).

$$\:{CBexp}_{i}={WHB}_{i}^{0.73}\times\:0.094$$

[Eq3] from Hatton et al.^1^, where CBexp_i_ is the expected carnivore biomass in year *i* and WHB_i_ is the wild herbivore biomass in year *i*

$$\:{CBexp}_{i}={HBexp}_{i}^{0.73}\times\:0.094$$

[Eq4] from Hatton et al.^1^, where CBexp_i_ is the expected carnivore biomass in year *i* and HBexp_i_ is the expected herbivore biomass in year *i* (from Eq. 1).

All data processing and analyses were carried out in the R environment 4.3.2^58^, using the packages sf^59^ and terra^60^ for spatial analysis and visualization.

## Electronic supplementary material

Below is the link to the electronic supplementary material.

Supplementary Material 1

## Data Availability

No primary data was generated in this study. All sources of data are cited in the manuscript.
