# Supplementary material for: The trophic distribution of biomass in ecosystems with co-occurring wildlife and livestock
Source: Sci Rep. 2025 Jan 9;15:1474. doi: 10.1038/s41598-025-85469-2 (PMC11718189; doi:10.1038/s41598-025-85469-2)
Supplement: Supplementary file 1 — Supplementary Material 1 [file 41598_2025_85469_MOESM1_ESM.docx]

## Supporting Information

**The trophic distribution of biomass in ecosystems with co-occurring wildlife and livestock**

**James D. M. Speed, Anna Sobocinski, Anders L. Kolstad, John D. C. Linnell, Erling J. Solberg, Jenny Mattisson, & Gunnar Austrheim**

Appendix A: Details for estimating carnivore biomass

Figure S1 Temporal trends in NPP across Norway

Figure S2: NPP mapped across Norway over time

Figure S3: Administrative borders of Norway

Figure S4: Herbivore densities across Norway over time

Figure S5: Carnivore densities across Norway over time

Figure S6 Backcast population trends of Norwegian carnivores

**Appendix A: Details for estimating carnivore biomass**

Historical densities of large carnivores in Norway were estimated using a back-calculation approach at the county level. We used the county boundaries prior to 2018. Carnivore ranges are large and can cross national boundaries; our population estimates are of individuals that spend at least part of the year in Norway. All large carnivore species greater than 10 kg were included, the grey wolf *Canis lupus*, brown bear *Ursus arctos*, Eurasian lynx *Lynx lynx* and wolverine *Gulo gulo*. This was based on the approach used by Mykrä and Pohja-Mykrä [1] to estimate Finnish carnivore densities back in time. The back-casting was based on hunting statistics data (as has been used also for wild herbivores in Norway [2, 3].We used the equation [1] to estimate the population of each species in the previous year (N_t-1_) based on the following year’s population (N_t_), the number of individuals killed by humans in that year (k_t_) along with the growth rate coefficient (r_t_) and a coefficient representing additional (non-anthropogenic) mortality (m_t_)

$$N_{t-1}=\frac{\left( N_{t}+k_{t} \right)}{\left[ \left( 1+m_{t} \right)e^{r_{t}} \right]}$$

The number of individuals killed by humans (k) was a combination of individuals hunted (and reported through the bounty system) plus other human-caused mortality including illegal hunting and vehicle collisions. These data were derived from historical reports, presenting the number of killed individuals per year and county (Statistics Norway 1846-2022, Table A1). Due to the bounty system employed for carnivore management in Norway up until the mid-20^th^ century [4] and the intensity of management regulation since the 1990’s, this data was well recorded, and assessed as reliable enough in our case as well as by Mykrä and Pohja-Mykrä [1]. Hunting data were based on calendar year prior to 1980 and hunting years (1^st^ April to 31st March) in the later years; in this period the number killed per hunting year was assigned to the calendar year that the hunting year ended in (31^st^ March). To account for additional hunting mortality which was not captured by bounty-derived statistics (i.e. animals fatally wounded but not retrieved), we followed Mykrä and Pohja-Mykrä [1] by adding a fixed 10% to all estimates of hunting mortality (k).

Other reported non-hunting mortality included carnivore individuals recorded as being killed by vehicles, illegal killings, animals found dead and animals killed without bounties being paid. These data were also extracted from the same reports as used above (but were absent between 1978 and 1987). These reports are certainly not comprehensive, but are minimum estimates.

In 1977, only national level data is available for the bear. The single bear reported in this year was assumed to have been felled in the county of Hedmark, based on the distribution of bears in the five years immediately preceding and following 1977. For the years 1984/85 - 1985/86, no data is available for the lynx. Therefore, k was assumed to be zero for these years. For the years 1846-1865, only combined data for Troms and Finnmark counties were presented. The combined mortality for these counties was split based on the mean ratio across these two counties over the twenty subsequent years (1866-1885).

For all counties except Oppland, Troms, and Finnmark, data was presented for the period 1866-70 (rather than annually). For Finnmark county, only combined data was available for the period 1933-1940 for the wolf and the wolverine. In some cases, the combined value was zero, therefore the yearly figures were automatically known to be zero. In cases of nonzero combined values, yearly data was derived by dividing the total figure evenly across the period.

The coefficient of (non-anthropogenic killing) mortality (m) and population growth rates (r) were estimated based on literature values, leaning towards Norwegian population estimates where possible (Table A2). A range of values for each were obtained. The initial population sizes of the large carnivores were based on Rovdata estimates for 2016.

We estimated the backcast populations iteratively for each species within each county. At each time step we performed a random drawing of values of m_t_ and r_t_ from a uniform distribution with range shown in Table A2. The backcasting model was bootstrapped (n = 100) for each species. We used the rounded mean of the bootstrapped values as the estimate of the population size in each year. The national level estimates are shown in Figure S2. The backcast estimates are reasonably consistent with population estimates from monitoring programmes over the most recent decades.

Population sizes were converted to biomass, as bear 200 kg, wolf 40 kg, lynx 20 kg and wolverine 13 kg as species level averages for Norwegian populations. Population biomass was converted to biomass density (kg km^-2^) based on the area of *utmark* (rangeland, unenclosed and unimproved land) within each county.

Table A1 Sources of human mortality data of carnivores in Norway

| **Year** | **Source** |
| --- | --- |
| 1846 – 1977 | Statistics Norway. Hunting Statistics 1846-1977. Oslo. Report No.: NOS A 955. https://www.ssb.no/a/histstat/nos/nos_a955.pdf |
| 1977 | Statistics Norway. Hunting Statistics 1977. Oslo. Report No.: NOS A 974. https://www.ssb.no/a/histstat/nos/nos_a974.pdf |
| 1978 | Statistics Norway. Hunting Statistics 1978. Oslo. Report No.: NOS B 44. https://www.ssb.no/a/histstat/nos/nos_b044.pdf |
| 1979 | Statistics Norway. Hunting Statistics 1979. Oslo. Report No.: NOS B 144. https://www.ssb.no/a/histstat/nos/nos_b144.pdf |
| 1980 | Statistics Norway. Hunting Statistics 1980. Oslo. Report No.: NOS B 219. https://www.ssb.no/a/histstat/nos/nos_b219.pdf |
| 1981 | Statistics Norway. Hunting Statistics 1981. Oslo. Report No.: NOS B 315. https://www.ssb.no/a/histstat/nos/nos_b315.pdf |
| 1982 | Statistics Norway. Hunting Statistics 1982. Oslo. Report No.: NOS B 417. https://www.ssb.no/a/histstat/nos/nos_b417.pdf |
| 1983 | Statistics Norway. Hunting Statistics 1983. Oslo. Report No.: NOS B 495. https://www.ssb.no/a/histstat/nos/nos_b495.pdf |
| 1984 | Statistics Norway. Hunting Statistics 1984. Oslo. Report No.: NOS B 567. https://www.ssb.no/a/histstat/nos/nos_b567.pdf |
| 1985 | Statistics Norway. Hunting Statistics 1985. Oslo. Report No.: NOS B 640. https://www.ssb.no/a/histstat/nos/nos_b640.pdf |
| 1986 | Statistics Norway. Hunting Statistics 1986. Oslo. Report No.: NOS B 721. https://www.ssb.no/a/histstat/nos/nos_b721.pdf |
| 1987 | Statistics Norway. Hunting Statistics 1987. Oslo. Report No.: NOS B 799. https://www.ssb.no/a/histstat/nos/nos_b799.pdf |
| 1987 – 2022 | Statistics Norway 2022. Registered mortality of large carnivores 1987-1988 – 2021-2022. Oslo, Norway https://www.ssb.no/en/statbank/table/03984/ |

Table A2 Range of r and m parameters for each species, as well as initial (2016) populations.

| **Species** | **r** | **m** | **N_2016_** | **References** |
| --- | --- | --- | --- | --- |
| Bear | 0.10 – 0.16 | 0.01 – 0.03 | 160 | [1, 5-8] |
| Wolf | 0.30 – 0.45 | 0.01 – 0.05 | 78 | [1, 9-15] |
| Lynx | 0.27 – 0.46 | 0.01 – 0.05 | 320 | [1, 16-19] |
| Wolverine | 0.28 – 0.39 | 0.01 – 0.05 | 386 | [1, 17, 20, 21] |

Table A3 Initial populations of each species used by county.

| **County** | **Bear** | **Wolf** | **Lynx** | **Wolverine** |
| --- | --- | --- | --- | --- |
| Finnmark | 35 | 0 | 17 | 39 |
| Troms | 10 | 1 | 34 | 51 |
| Nordland | 3 | 0 | 35 | 75 |
| Nord-Trøndelag | 29 | 0 | 21 | 25 |
| Sør-Trøndelag | 2 | 0 | 19 | 19 |
| Møre og Romsdal | 0 | 0 | 17 | 25 |
| Sogn og Fjordane | 0 | 0 | 0 | 0 |
| Oppland | 0 | 0 | 11 | 50 |
| Hedmark | 46 | 68 | 46 | 65 |
| Hordaland | 0 | 0 | 0 | 0 |
| Buskerud | 0 | 0 | 15 | 0 |
| Akershus | 0 | 10 | 0 | 0 |
| Telemark | 0 | 0 | 20 | 0 |
| Vestfold | 0 | 0 | 0 | 0 |
| Østfold | 0 | 3 | 18 | 0 |
| Rogaland | 0 | 0 | 0 | 0 |
| Vest-Agder | 0 | 0 | 0 | 0 |
| Aust-Agder | 0 | 0 | 2 | 0 |

**Appendix A References**

1. Mykrä, S. and M. Pohja-Mykrä. *Back-calculation of large carnivore populations in Finland in 1865–1915*. in *Annales Zoologici Fennici*. 2015. BioOne.

2. Austrheim, G., E.J. Solberg, and A. Mysterud, *Spatio-temporal distribution of large herbivores in Norway from 1949 to 1999: Has decreased grazing by domestic herbivores been countered by increased browsing by cervids?* Wildlife Biology, 2011. **17**: p. 1-13.

3. Speed, J.D.M., et al., *Long-term changes in northern large-herbivore communities reveal differential rewilding rates in space and time.* PLoS ONE, 2019. **14(5)**: p. e0217166.

4. E., S., *Tallenes fortellinger [The tale of statistics]*. 2001: Samfunnsspeilet/Statistisk sentralbyrå, Oslo.

5. Kojola, I., *Biology of the bear and the current status of the bear population*. 2007, Ministry of Agriculture and Forestry: Helsinki.

6. Kojola, I. and S. Heikkinen, *The structure of the expanded brown bear population at the edge of the Finnish range.* Ann Zool Fennici, 2006. **43**: p. 258–262.

7. Sæther, B.-E., et al., *Assessing the Viability of Scandinavian Brown Bear, Ursus arctos, Populations: The Effects of Uncertain Parameter Estimates.* Oikos, 1998. **83**: p. 403–416.

8. Wiegand, T., et al., *Assessing the risk of extinction for the brown bear (Ursus arctos) in the cordillera cantabrica, Spain.* Ecol Monogr, 1998. **68**: p. 539–570.

9. Creel, S. and J.J. Rotella, *Meta-analysis of relationships between human offtake, total mortality and population dynamics of gray wolves (Canis lupus).* PLoS One, 2010. **5**.

10. Fuller, T.K., L.D. Mech, and J.F. Cochrane, *Wolf Population Dynamics*, in *Wolves: Behavior, Ecology, and Conservation*, L.D. Mech and L. Boitani, Editors. 2003, University of Chicago Press: Chicago. p. 161–191.

11. Mech, L.D., *Managing Minnesota’s Recovered Wolves.* Wildl Soc Bull, 2001. **29**: p. 70–77.

12. Mech, L.D. and J. Fieberg, *Growth rates and variances of unexploited wolf populations in dynamic equilibria.* Wildl Soc Bull, 2015. **39**: p. 41–48.

13. Sime, C.A., et al., *Montana gray wolf conservation and management 2010 annual report*. 2011, Montana Fish, Wildlife & Parks: Helena.

14. Wielgus, R.B. and K.A. Peebles, *Effects of wolf mortality on livestock depredations.* PLoS One, 2014. **9**: p. e113505.

15. Wiles, G.J., H.L. Allen, and G.E. Hayes, *Wolf conservation and management plan for Washington*. 2011, Washington Department of Fish and Wildlife: Olympia.

16. Andrén, H., et al., *Survival rates and causes of mortality in Eurasian lynx (Lynx lynx) in multi-use landscapes.* Biol Conserv, 2006. **131**: p. 23–32.

17. Mills, L.S., M. Hebblewhite, and D.R. Eacker, *Bayesian Population Viability Analysis for Lynx and Wolverine in Scandinavia*. 2018, Swedish Environmental Protection Agency: Stockholm.

18. Palmero, S., et al., *Demography of a Eurasian lynx (Lynx lynx) population within a strictly protected area in Central Europe.* Sci Rep, 2021. **11**.

19. Slough, B.G. and G. Mowat, *Lynx Population Dynamics in an Untrapped Refugium.* J Wildl Manage, 1996. **60**: p. 946–961.

20. Krebs, J., et al., *Synthesis of survival rates and causes of mortality in North American wolverines.* J Wildl Manage, 2004.

21. Persson, J., *Population ecology of Scandinavian wolverines*. 2003, Swedish University of Agricultural Sciences: Umeå.

22. R Core Team, *R: A language and environment for statistical computing*. 2024, R Foundation for Statistical Computing <https://www.r-project.org/>.

23. Hijmans, R., *terra: Spatial Data Analyais. R package version 1.7-78.* <https://CRAN.R-project.org/package=terra>, 2024.

24. Pebesma, E.J., *Simple features for R: standardized support for spatial vector data.* R Journal, 2018. **10**(1): p. 439 <https://doi.org/10.32614/RJ-2018-009>.

Table S1. Source year for biomass data per trophic level. Data is presented as the year in the “assigned year” column. Herbivore biomass densities were estimated for the years 1907, 1917, (all) 1927 (wild herbivores only), 1929 (livestock), 1938 (wild herbivores), 1939 (livestock), and all species every 10^th^ year from 1949 to 2009 and 2015. This selection of years was due to data availability. The livestock and wild herbivore datasets from 1927 and 1929 and 1938 and 1939 were combined to estimate the total herbivore biomass in the latter year of each pair.

| **Assigned year** | **Plant productivity** | **Wild Herbivore biomass** | **Livestock biomass** | **Carnivore biomass** |
| --- | --- | --- | --- | --- |
| 2015 | 2015 | 2015 | 2015 | 2015 |
| 2009 | 2009 | 2009 | 2009 | 2009 |
| 1999 | 2000 | 1999 | 1999 | 1999 |
| 1989 |  | 1989 | 1989 | 1989 |
| 1979 |  | 1979 | 1979 | 1969 |
| 1969 |  | 1969 | 1969 | 1969 |
| 1959 |  | 1959 | 1959 | 1959 |
| 1949 |  | 1949 | 1949 | 1949 |
| 1939 |  | 1938 | 1939 | 1939 |
| 1929 |  | 1927 | 1929 | 1927 |
| 1917 |  | 1917 | 1919 | 1917 |
| 1907 |  | 1907 | 1909 | 1907 |
| 1896 |  |  |  | 1896 |
| 1886 |  |  |  | 1886 |
| 1876 |  |  |  | 1876 |
| 1866 |  |  |  | 1866 |
| 1856 |  |  |  | 1856 |
| 1846 |  |  |  | 1846 |


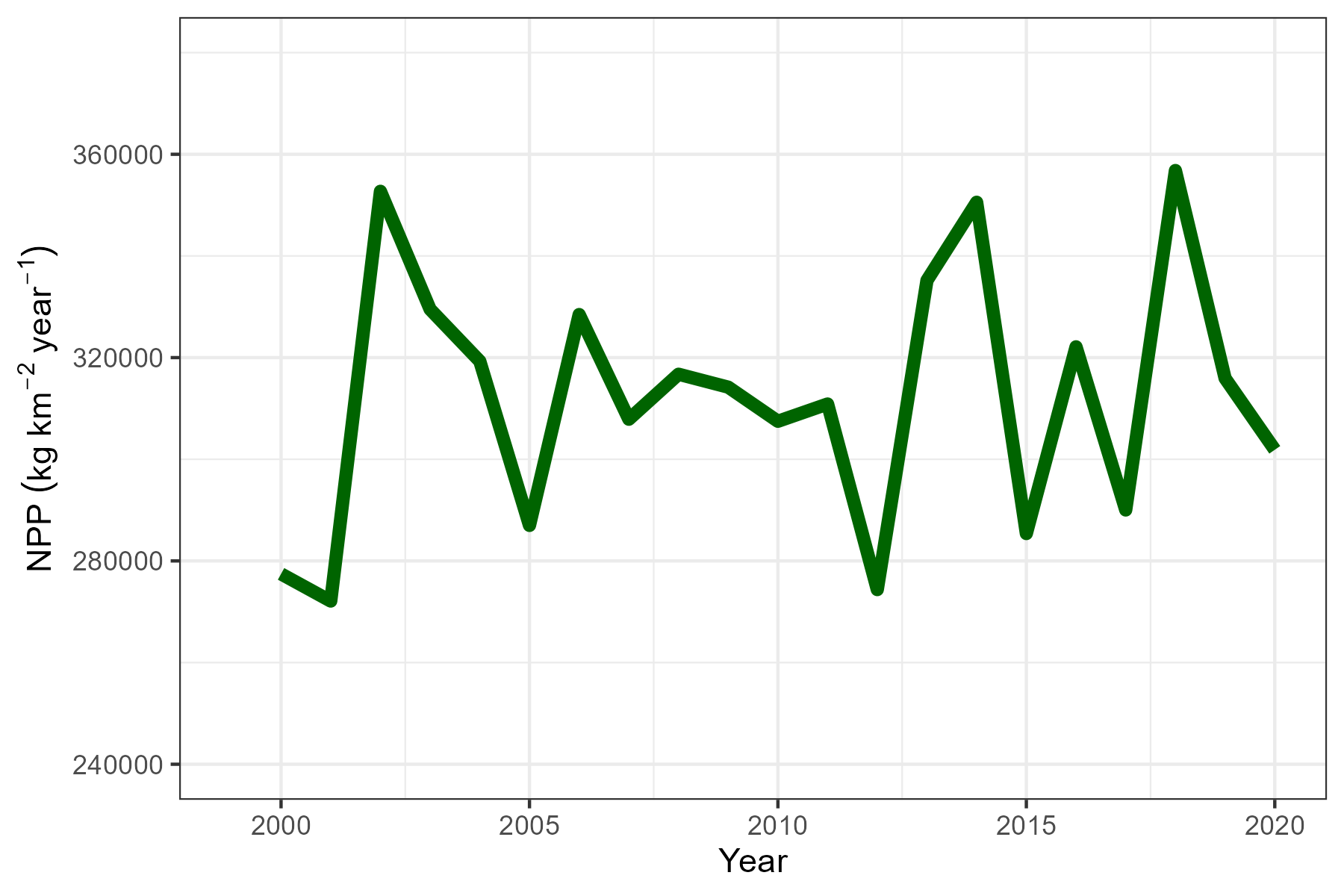


Figure S1 Temporal trends in mean net primary productivity across Norway


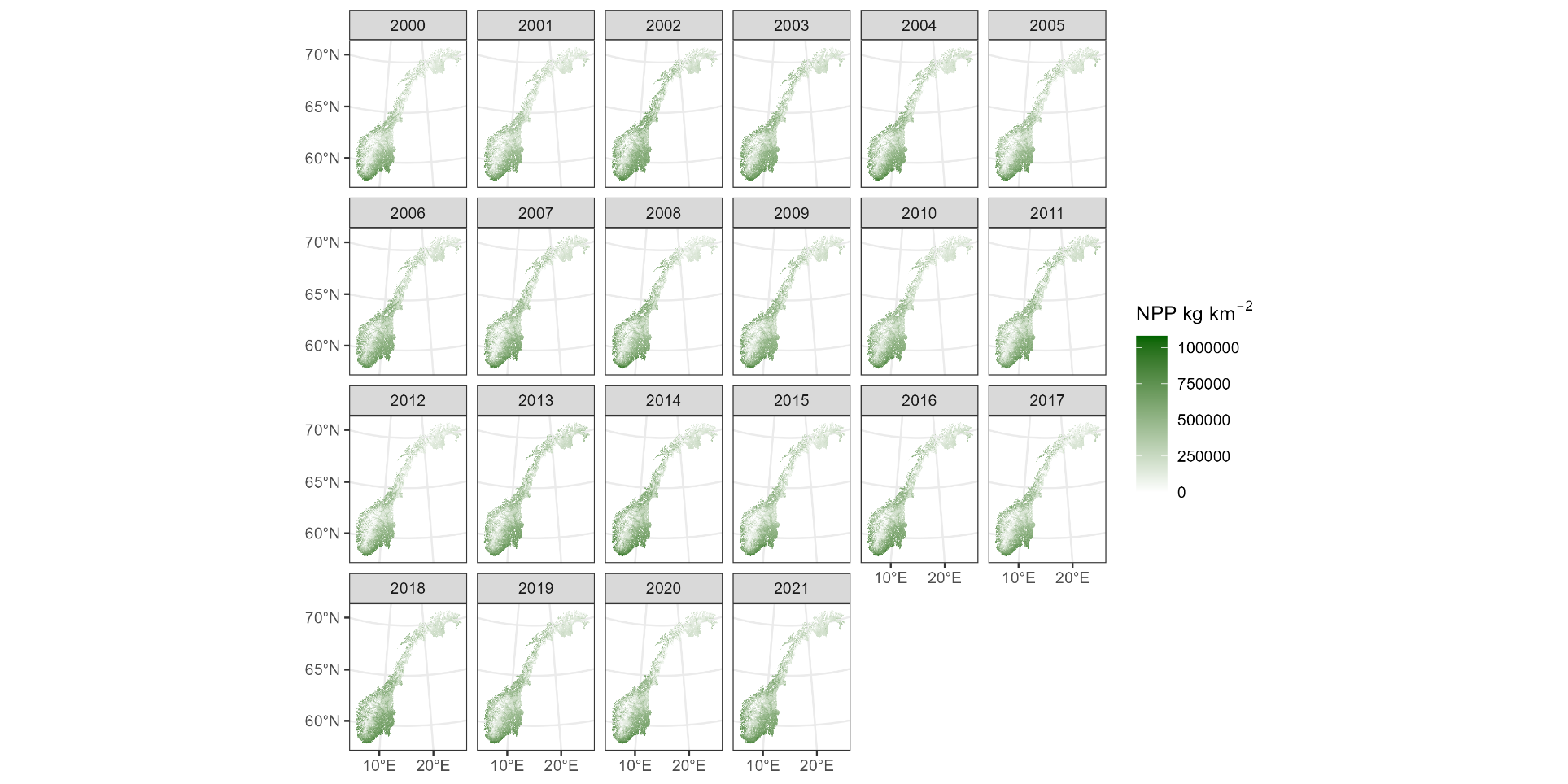


Figure S2 Net primary productivity (kg km^-2^) across Norway, yearly between 2000 and 2021. Maps created in R [22], using the package terra [23].


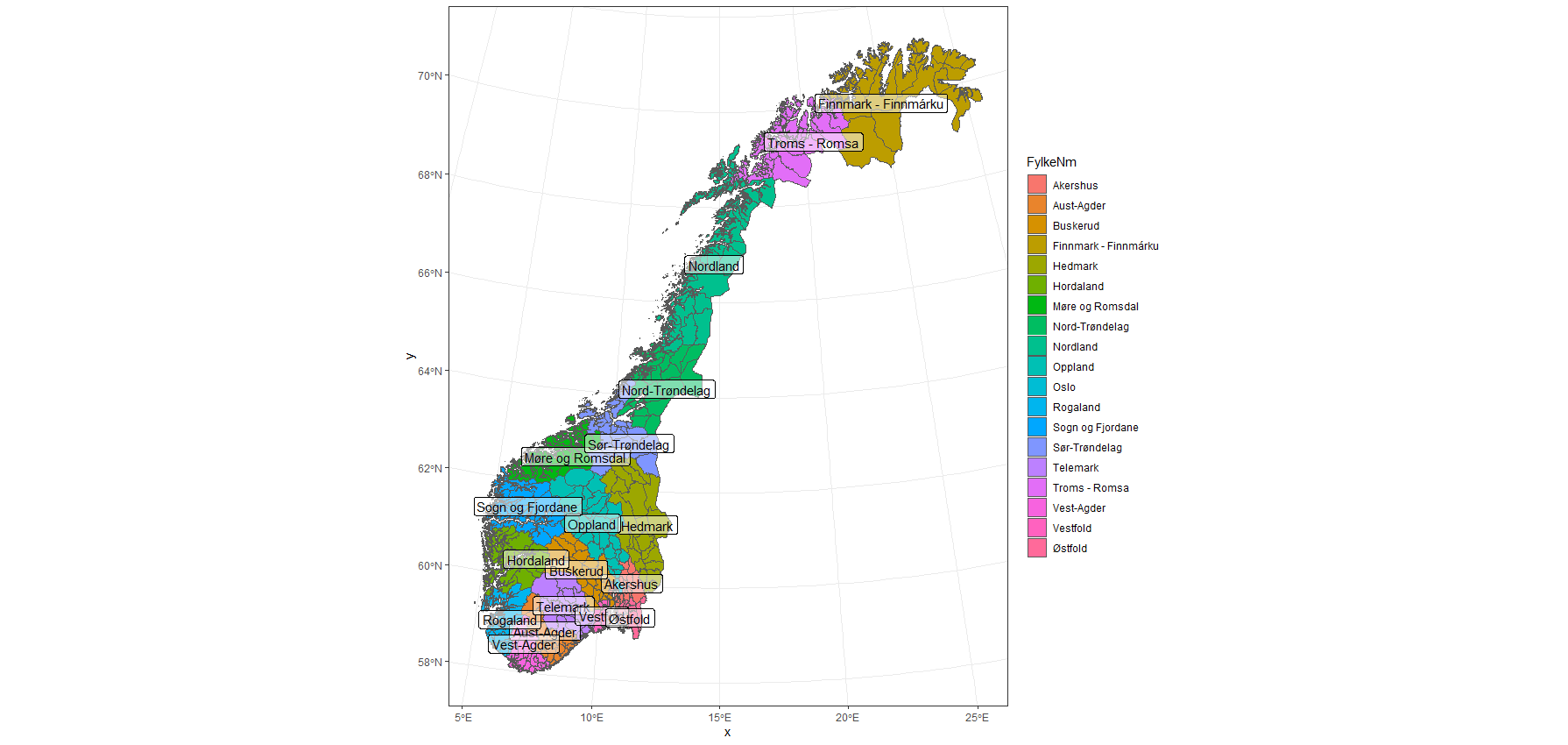


Figure S3 Administrative boundaries of Norway used in the study. Municipality borders are shown as polygons, coloured (and labelled) by county. Administrative regions and borders were those in existence in 2016. Map created in R [22], using the package sf [24].


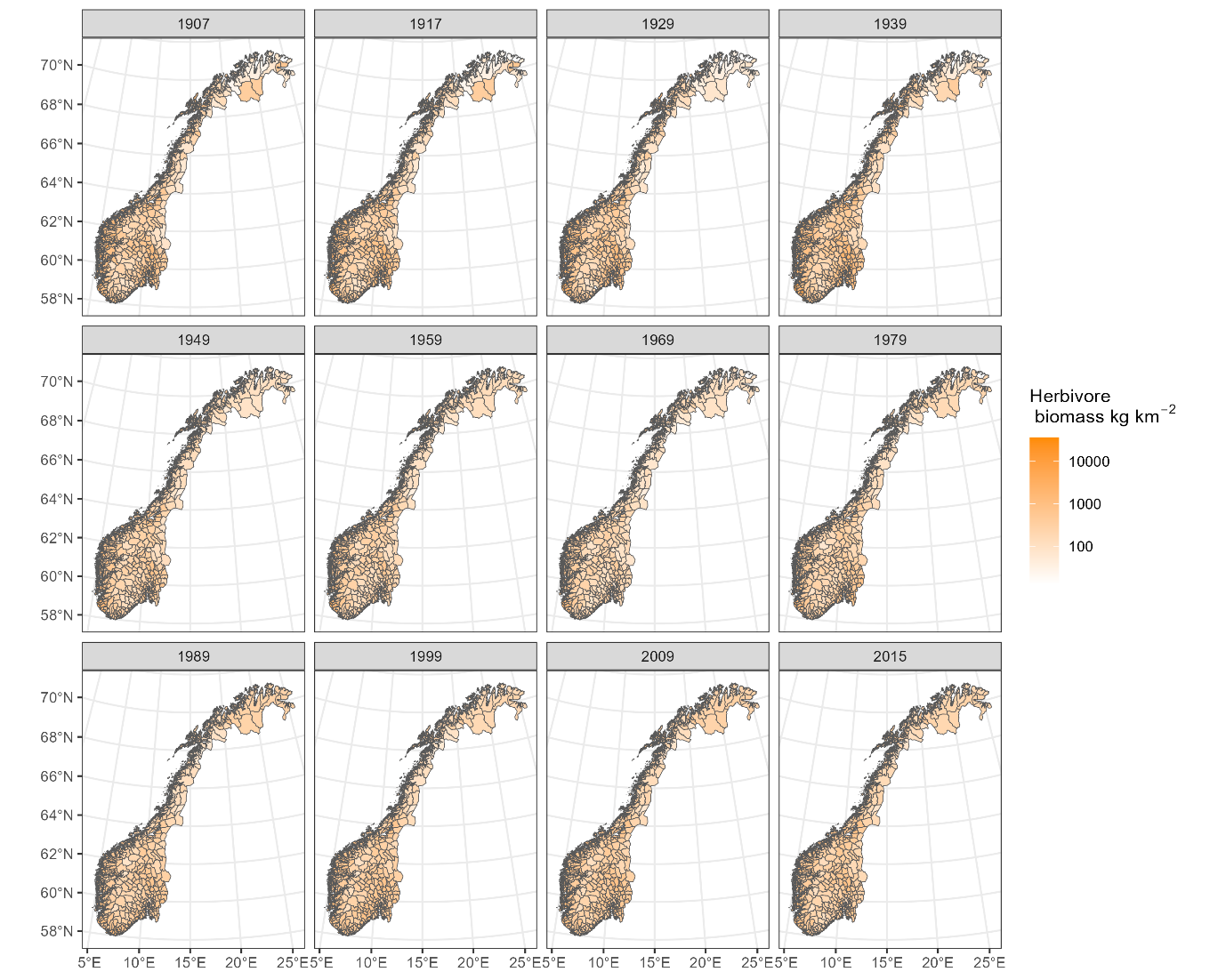


Figure S4a Total herbivore biomass density (kg km^-2^) across Norway in selected years. Note that colour scales vary between species or species groups. The figures are suitable for temporal comparison within species but less suitable for comparison between species. Maps created in R [22], using the package sf [24].


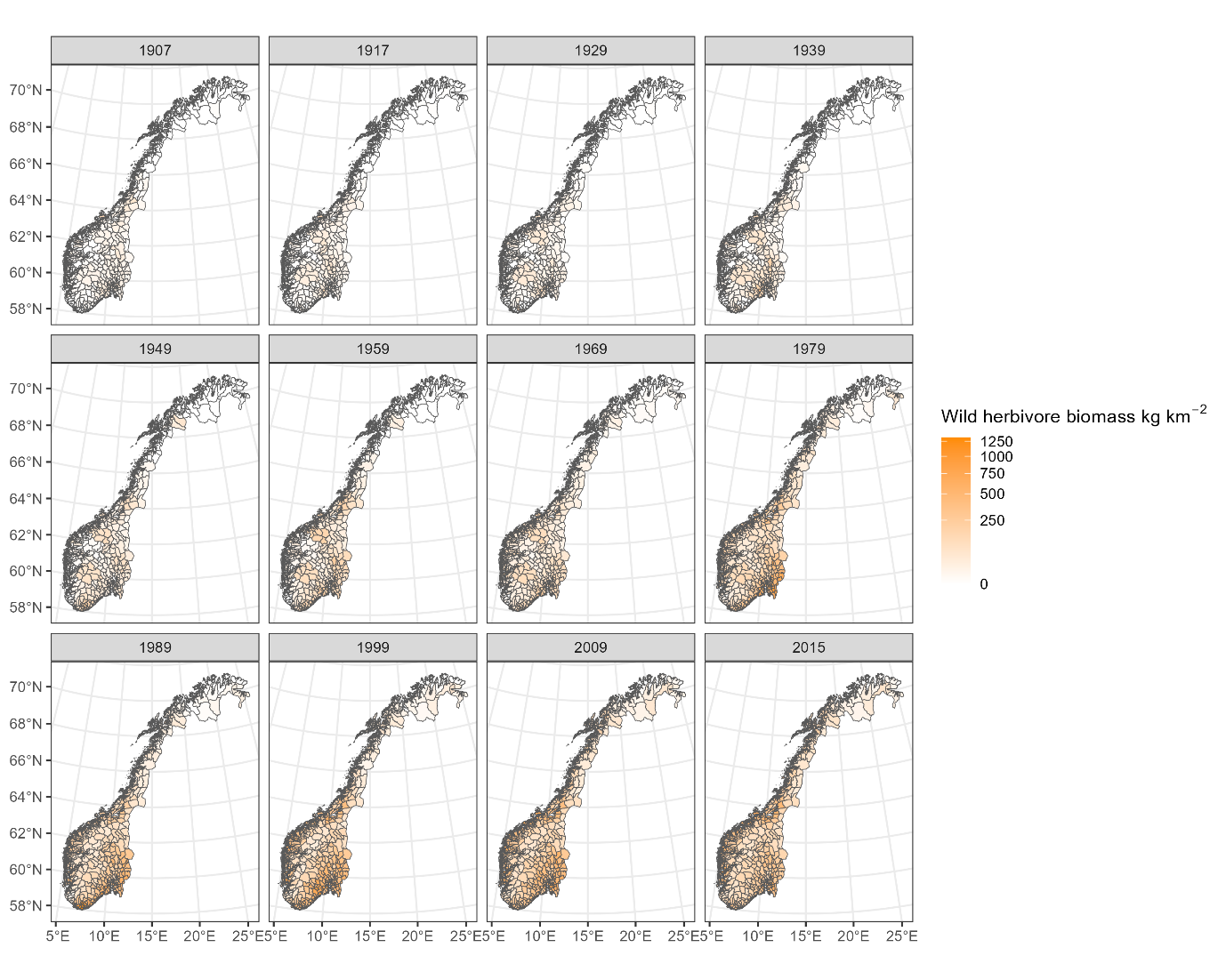


Figure S4b Wild herbivore biomass density (kg km^-2^) across Norway in selected years. Note that colour scales vary between species. The figures are suitable for temporal comparison within species but less suitable for comparison between species. Maps created in R [22], using the package sf [24].


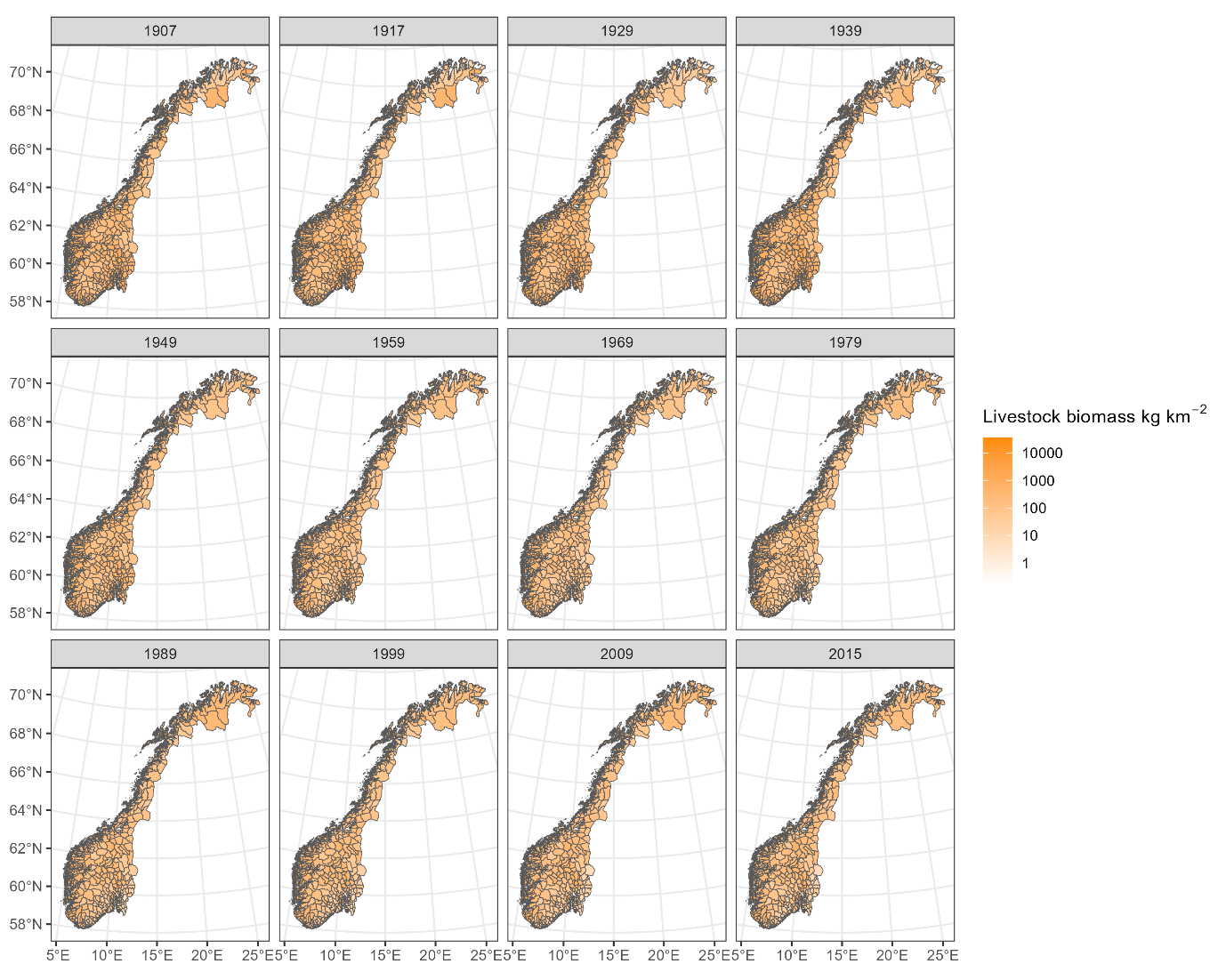


Figure S4c Livestock herbivore biomass density (kg km^-2^) across Norway in selected years. Note that colour scales vary between species. The figures are suitable for temporal comparison within species but less suitable for comparison between species. Maps created in R [22], using the package sf [24].


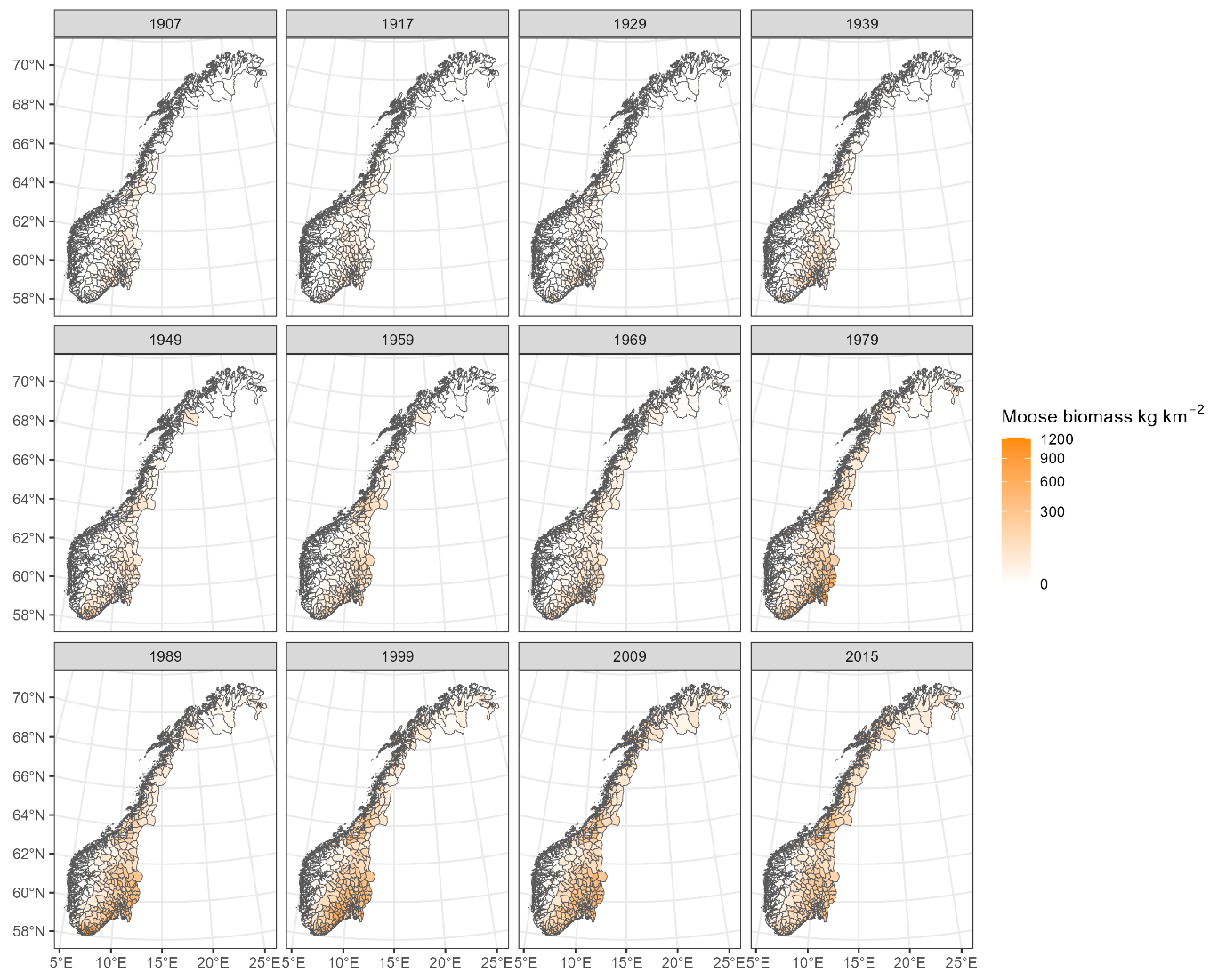


Figure S4d Moose biomass density (kg km^-2^) across Norway in selected years. Note that colour scales vary between species. The figures are suitable for temporal comparison within species but less suitable for comparison between species. Map created in R [22], using the package sf [24].


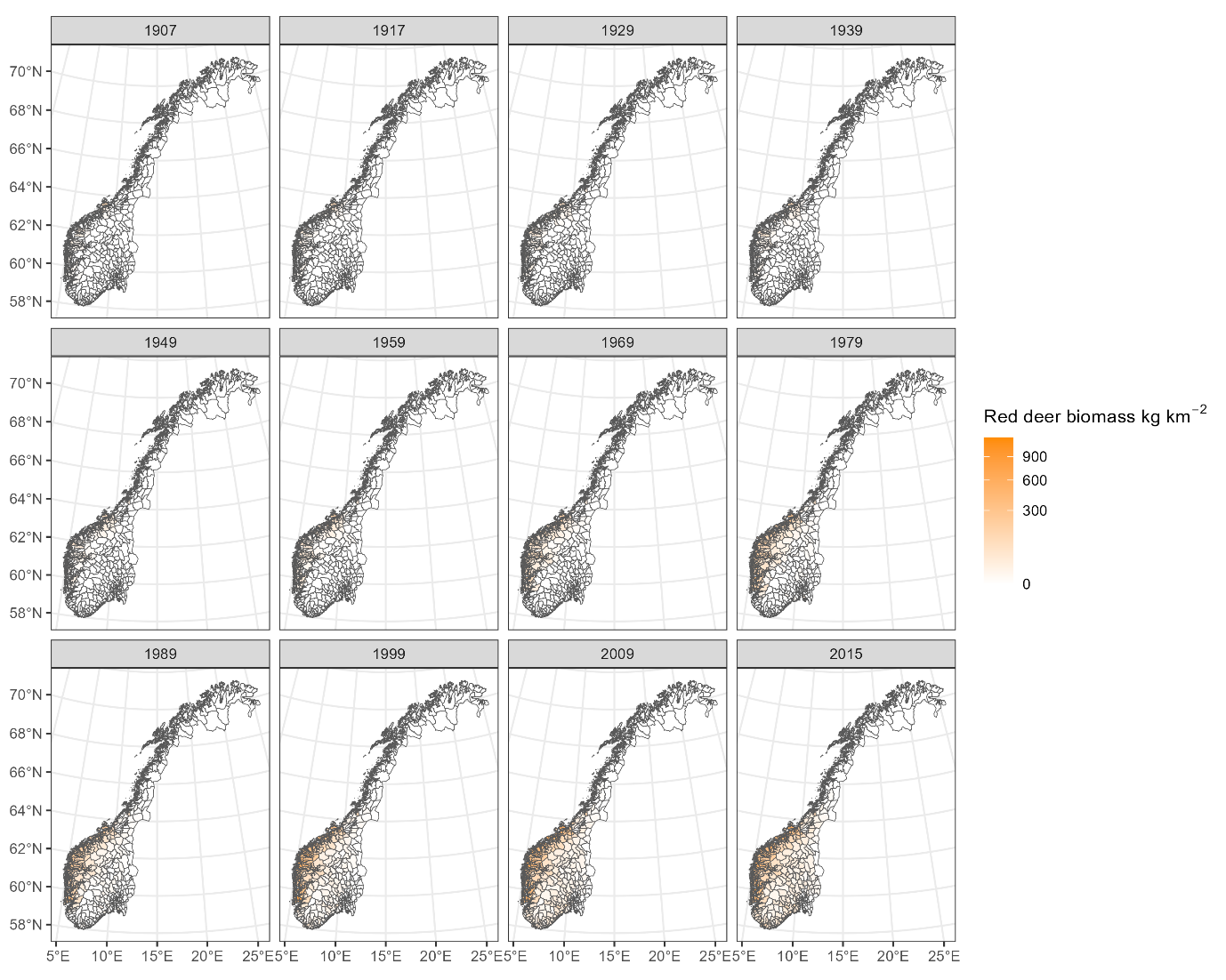


Figure S4e Red deer biomass density (kg km^-2^) across Norway in selected years. Note that colour scales vary between species. The figures are suitable for temporal comparison within species but less suitable for comparison between species. Map created in R [22], using the package sf [24].


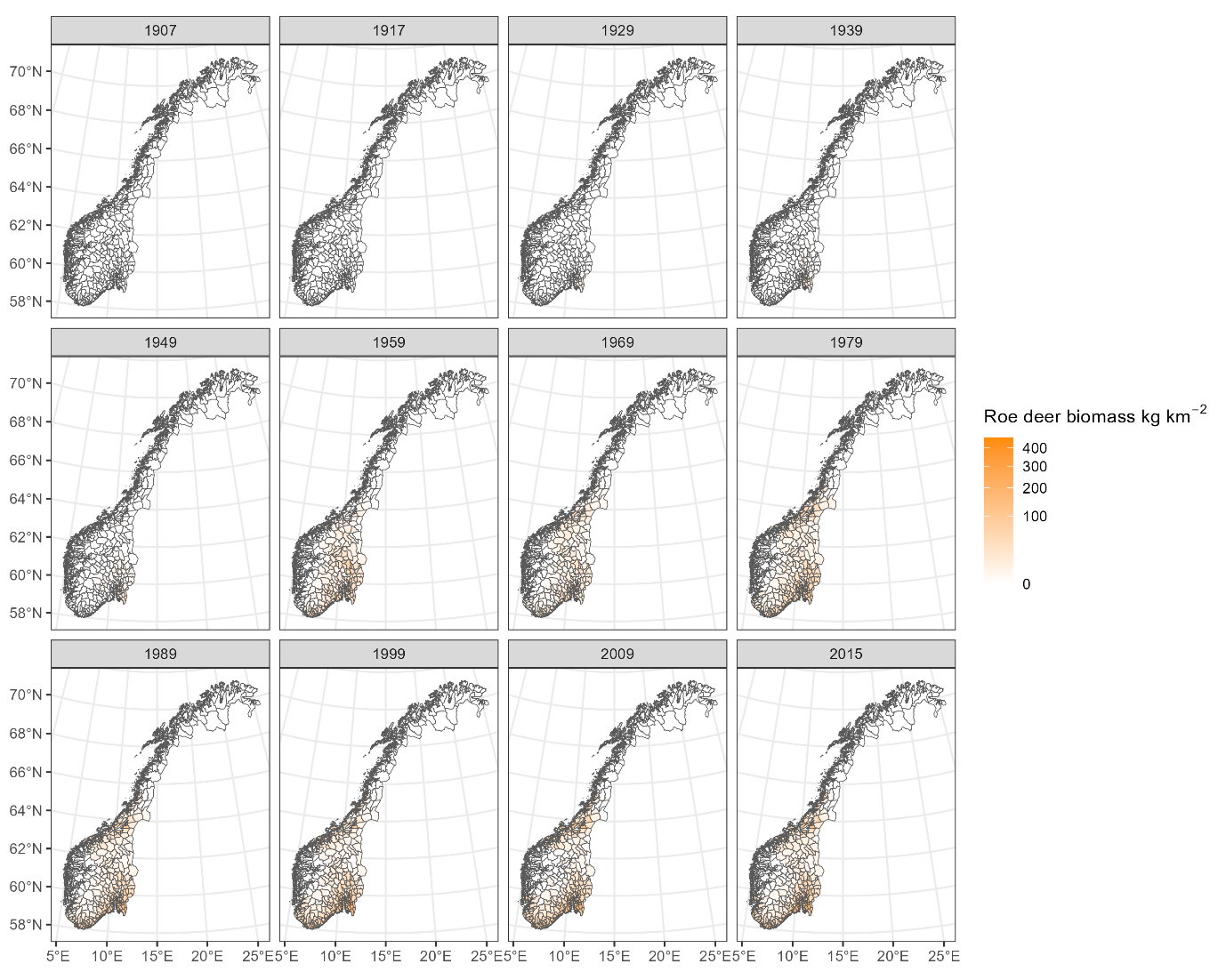


Figure S4f Roe deer biomass density (kg km^-2^) across Norway in selected years. Note that colour scales vary between species. The figures are suitable for temporal comparison within species but less suitable for comparison between species. Map created in R [22], using the package sf [24].


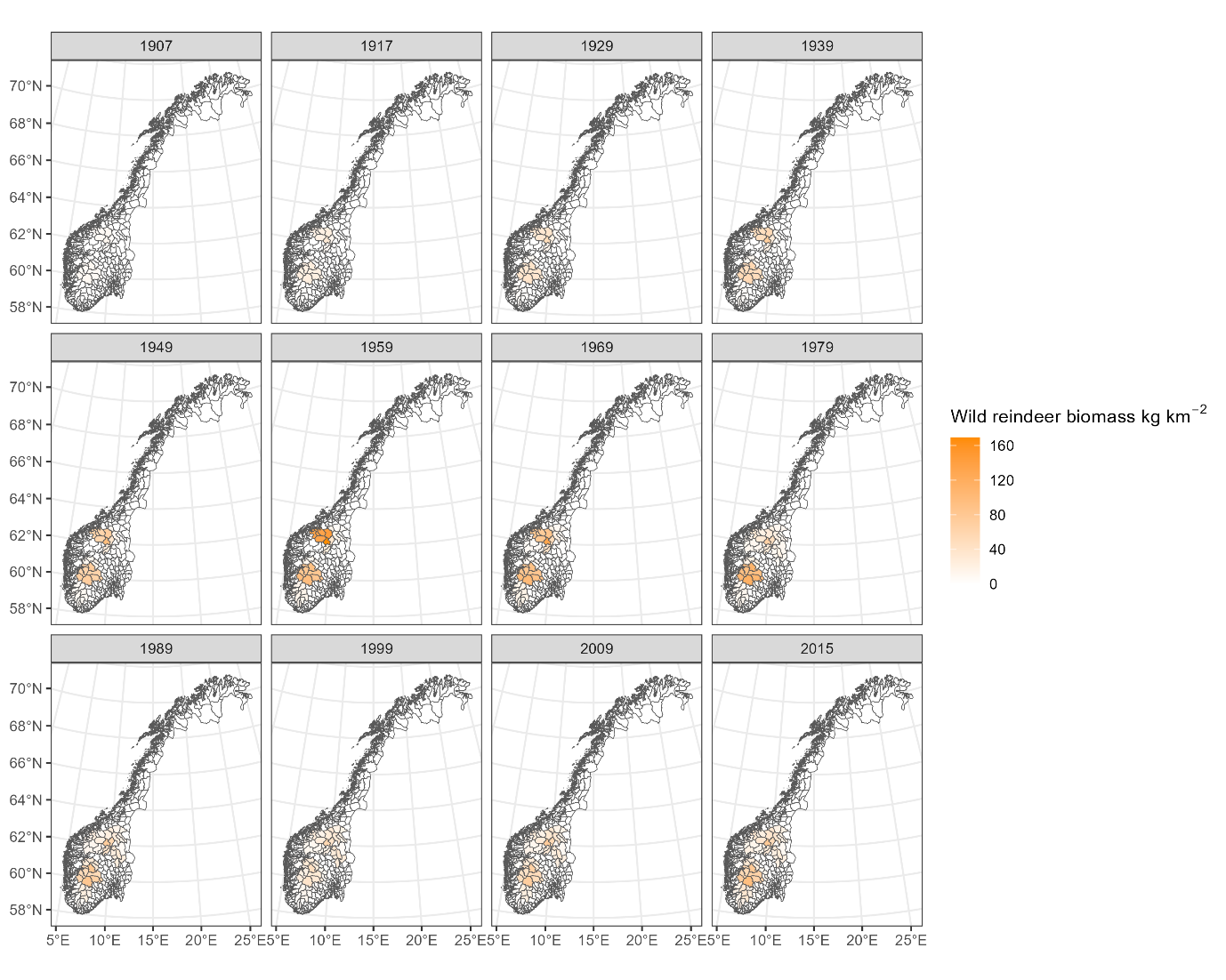


Figure S4g Wild reindeer biomass density (kg km^-2^) across Norway in selected years. Note that colour scales vary between species. The figures are suitable for temporal comparison within species but less suitable for comparison between species. Map created in R [22], using the package sf [24].


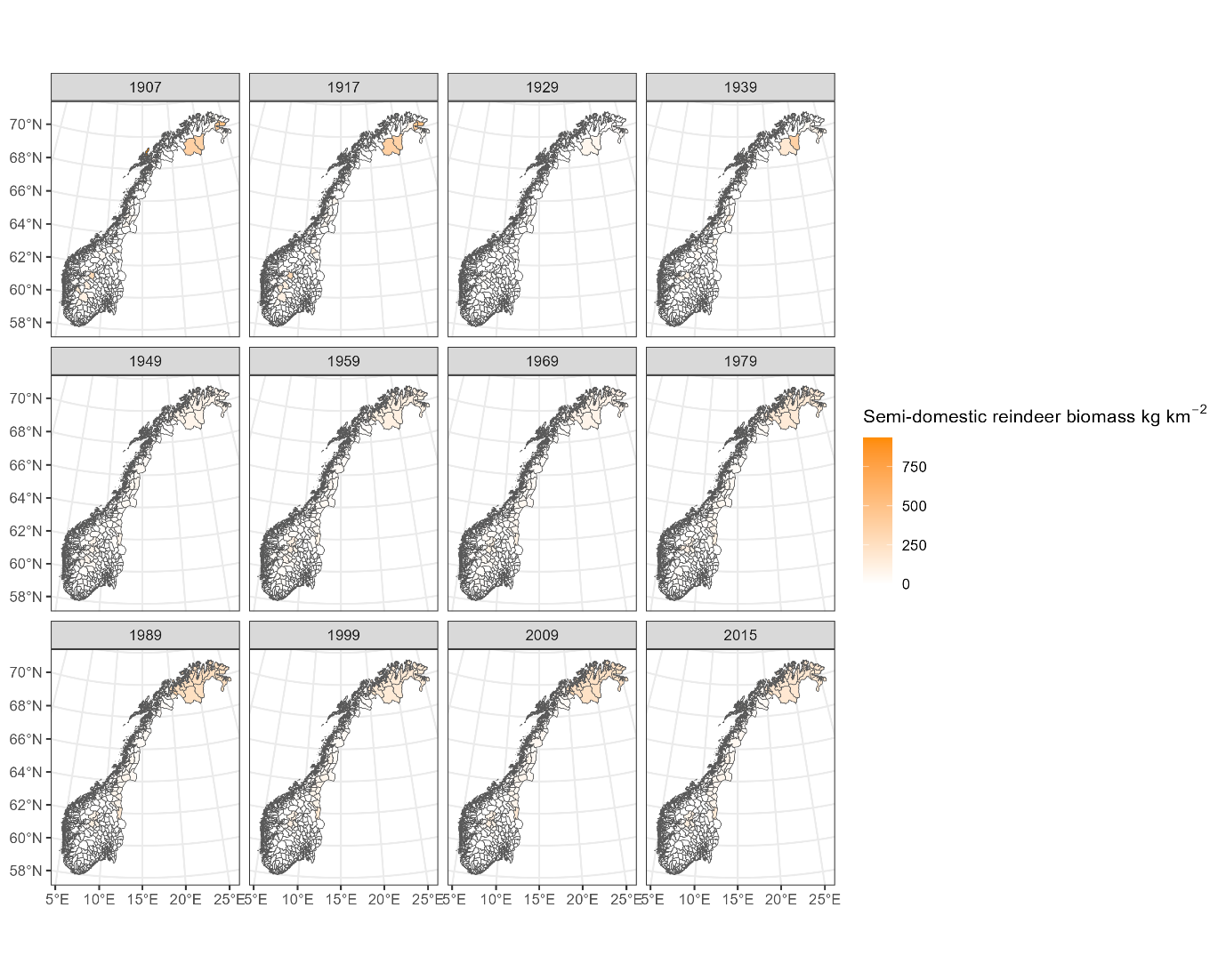


Figure S4h Semi-domestic reindeer biomass density (kg km^-2^) across Norway in selected years. Note that colour scales vary between species. The figures are suitable for temporal comparison within species but less suitable for comparison between species. Map created in R [22], using the package sf [24].


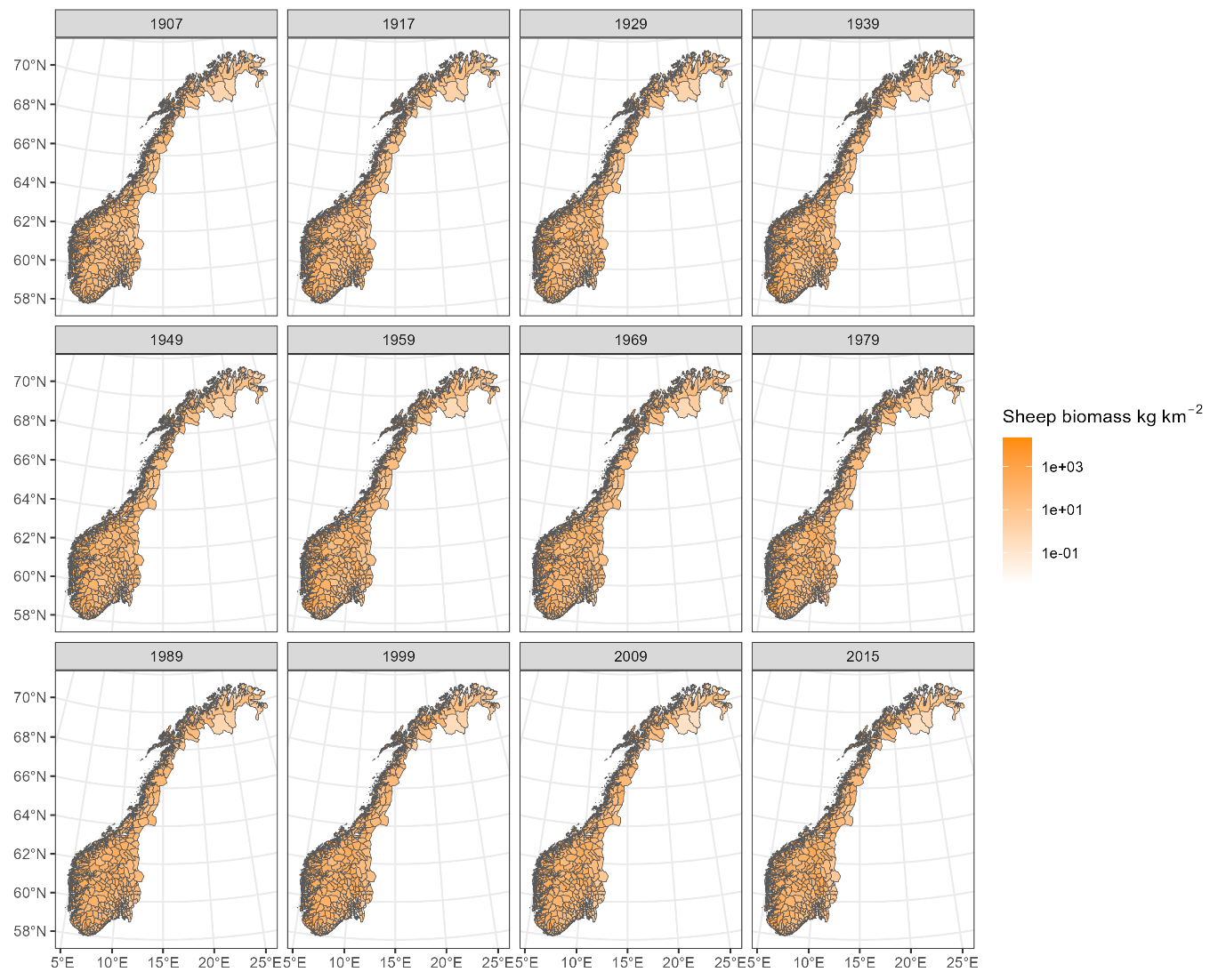


Figure S4i Sheep biomass density (kg km^-2^) across Norway in selected years. Note that colour scales vary between species. The figures are suitable for temporal comparison within species but less suitable for comparison between species. Map created in R [22], using the package sf [24].


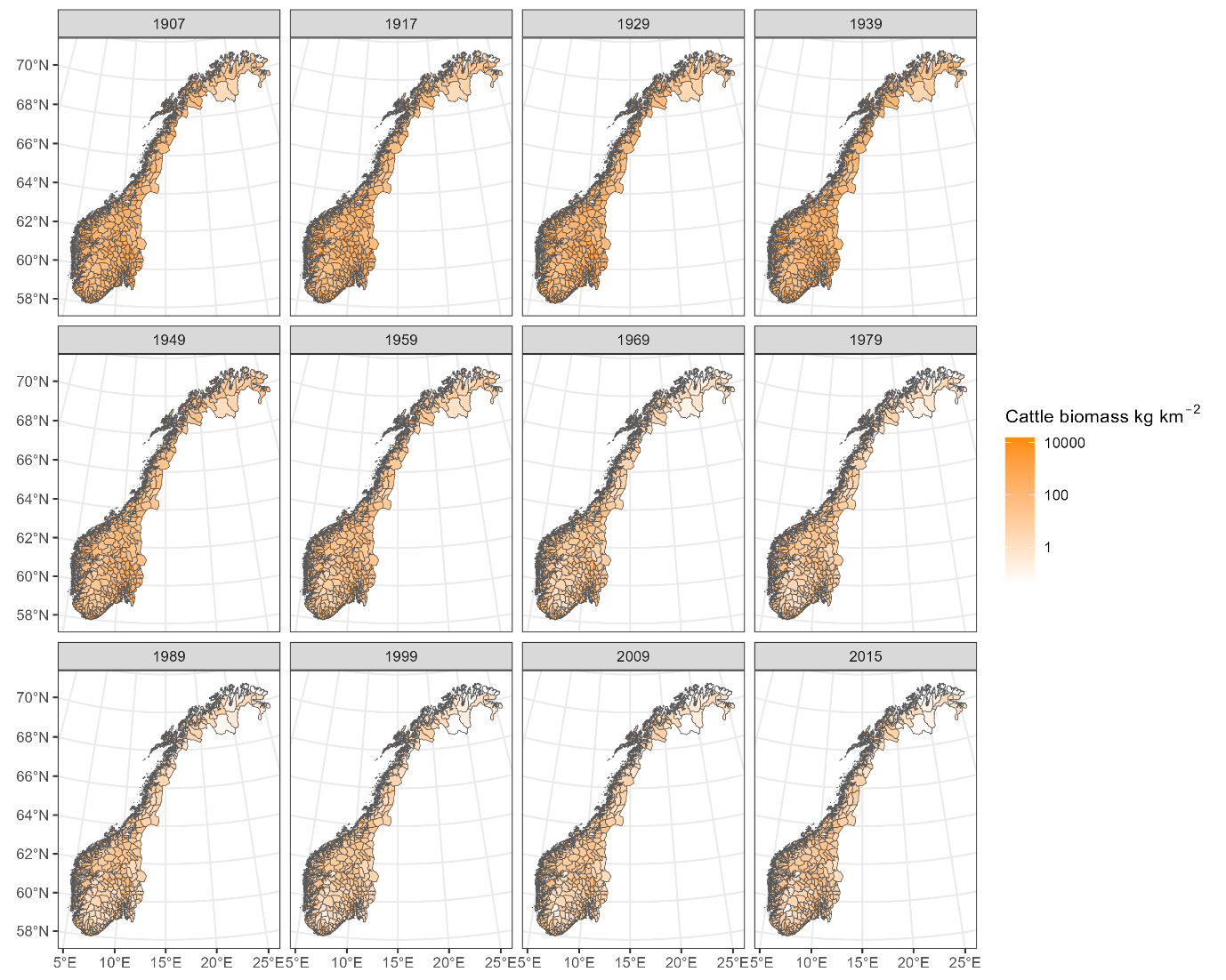


Figure S4j Cattle biomass density (kg km^-2^) across Norway in selected years. Note that colour scales vary between species. The figures are suitable for temporal comparison within species but less suitable for comparison between species. Map created in R [22], using the package sf [24].


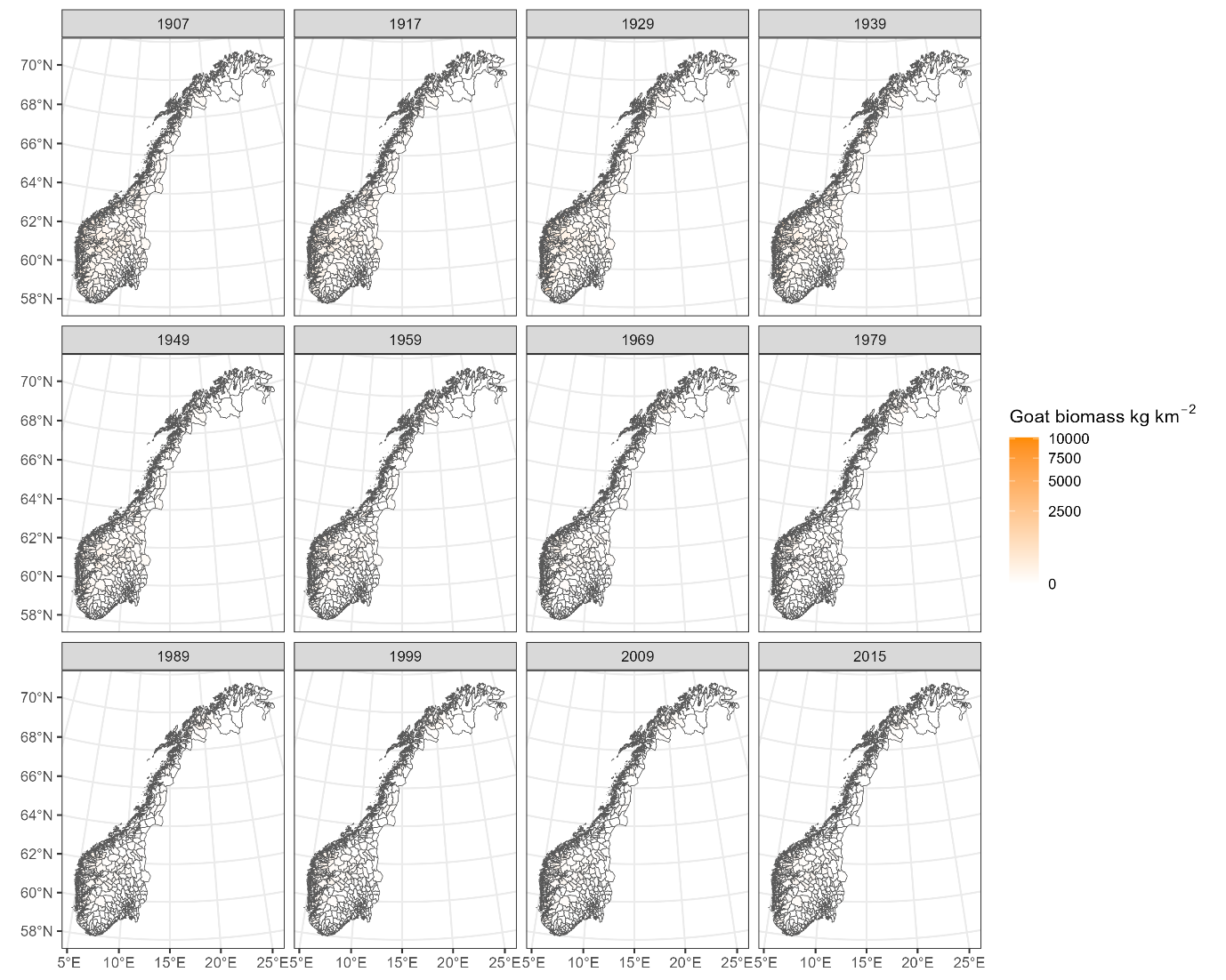


Figure S4k Goat biomass density (kg km^-2^) across Norway in selected years. Note that colour scales vary between species. The figures are suitable for temporal comparison within species but less suitable for comparison between species. Map created in R [22], using the package sf [24].


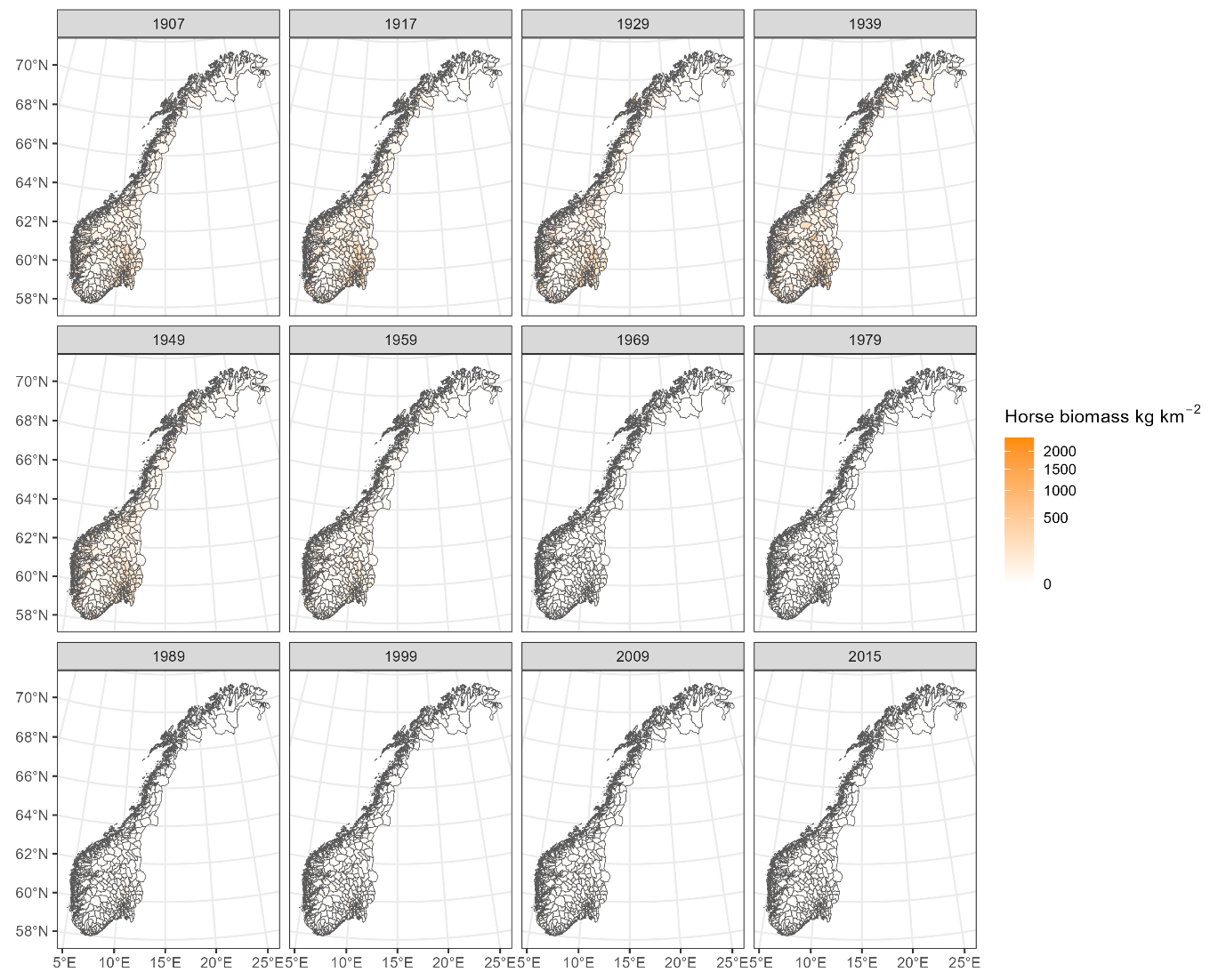


Figure S4l Horse herbivore biomass density (kg km^-2^) across Norway in selected years. Note that colour scales vary between species. The figures are suitable for temporal comparison within species but less suitable for comparison between species. Map created in R [22], using the package sf [24].


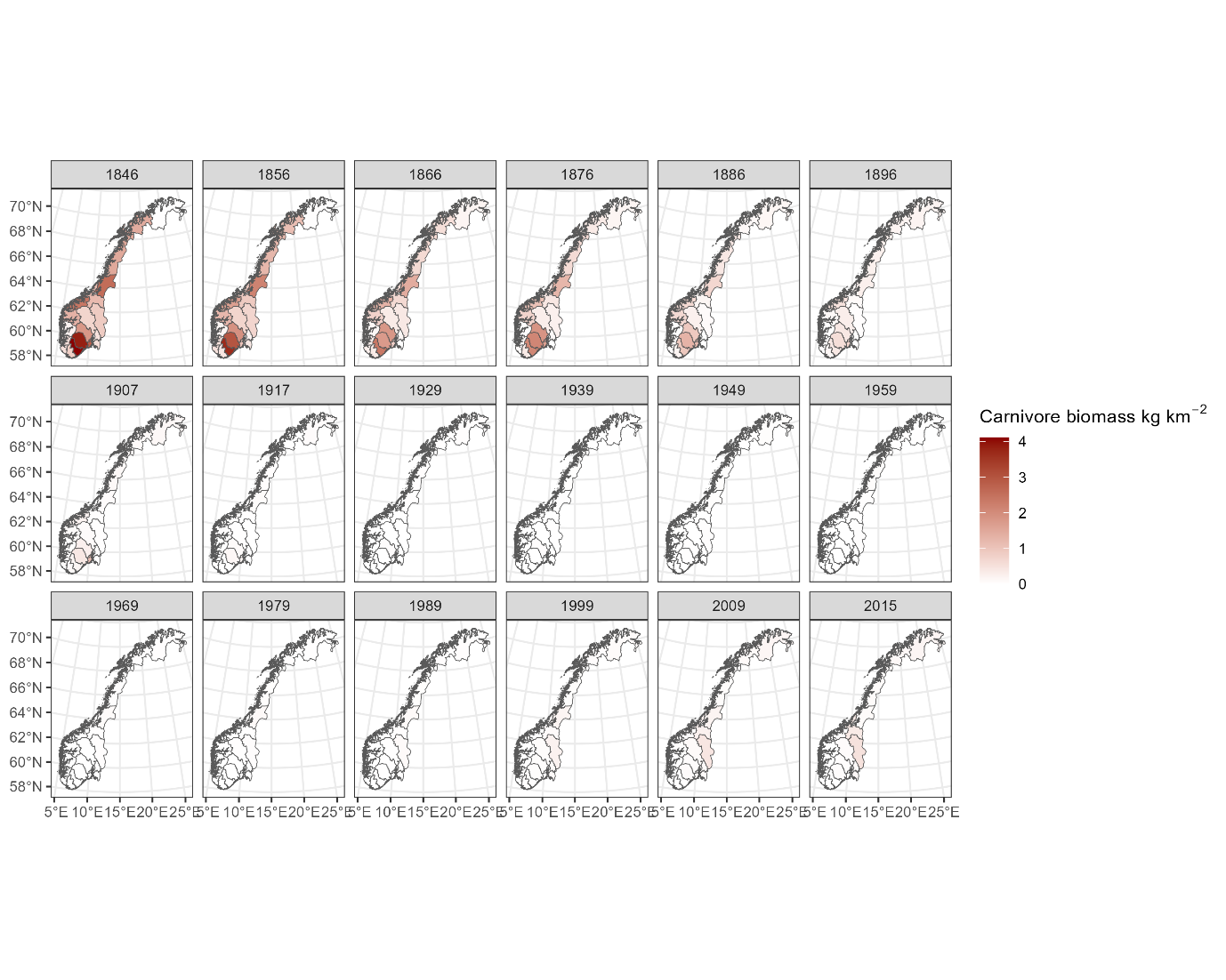


Figure S5a Total carnivore biomass density (kg km^-2^) across Norway in selected years. Note that colour scales vary between species. The figures are suitable for temporal comparison within species but less suitable for comparison between species. Map created in R [22], using the package sf [24].


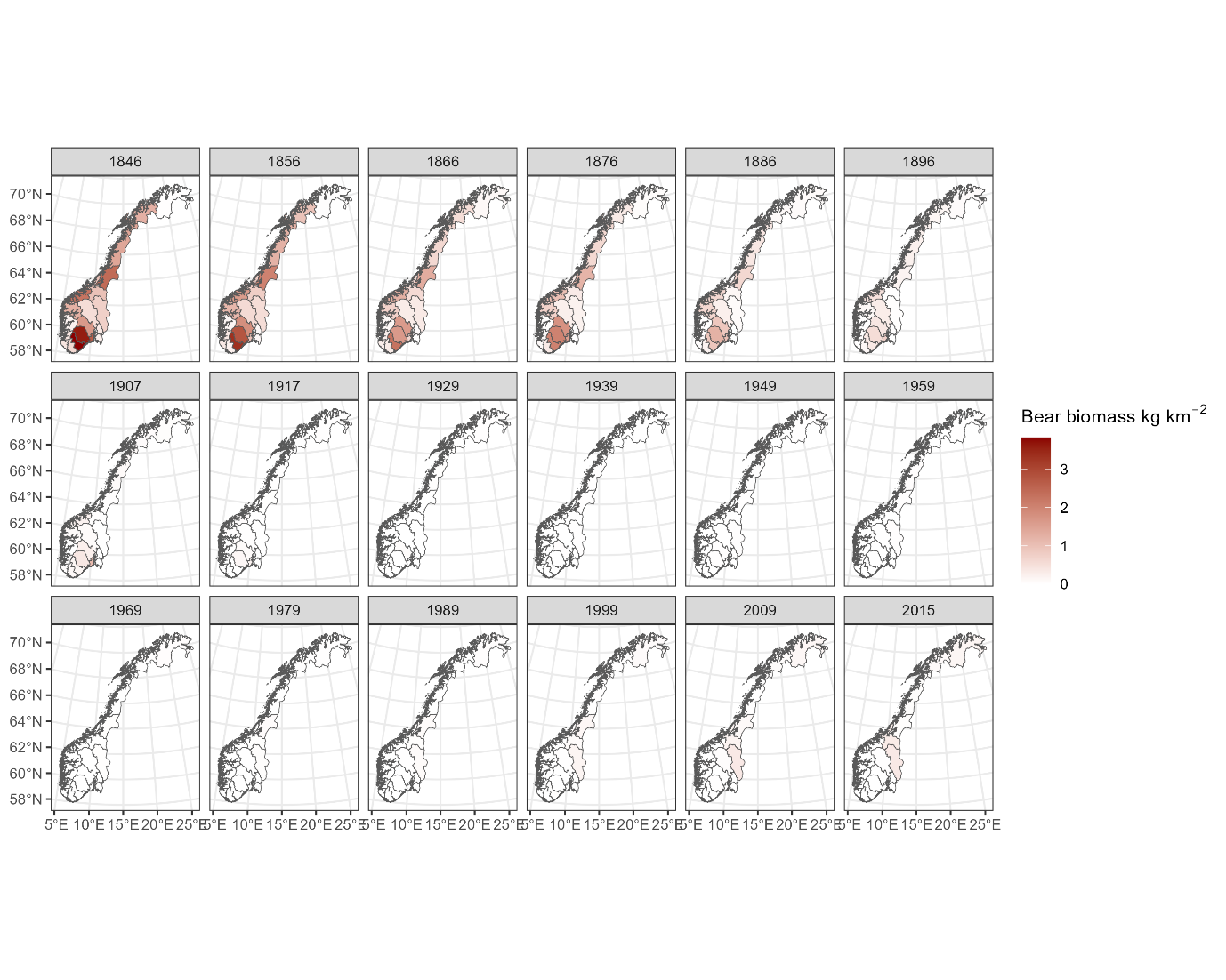


Figure S5b Brown bear biomass density (kg km^-2^) across Norway in selected years. Note that colour scales vary between species. The figures are suitable for temporal comparison within species but less suitable for comparison between species. Map created in R [22], using the package sf [24].


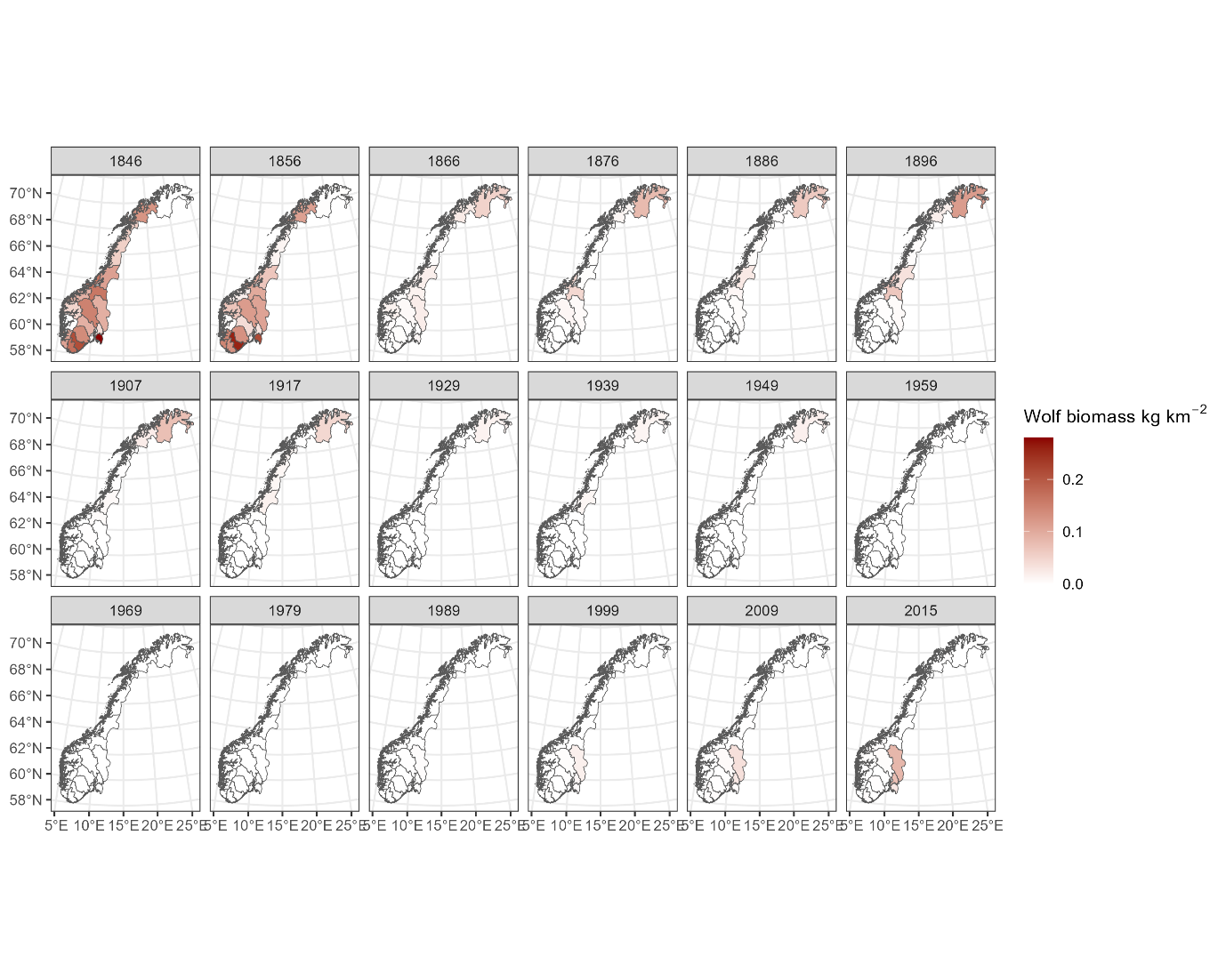


Figure S5c Wolf biomass density (kg km^-2^) across Norway in selected years. Note that colour scales vary between species. The figures are suitable for temporal comparison within species but less suitable for comparison between species. Map created in R [22], using the package sf [24].


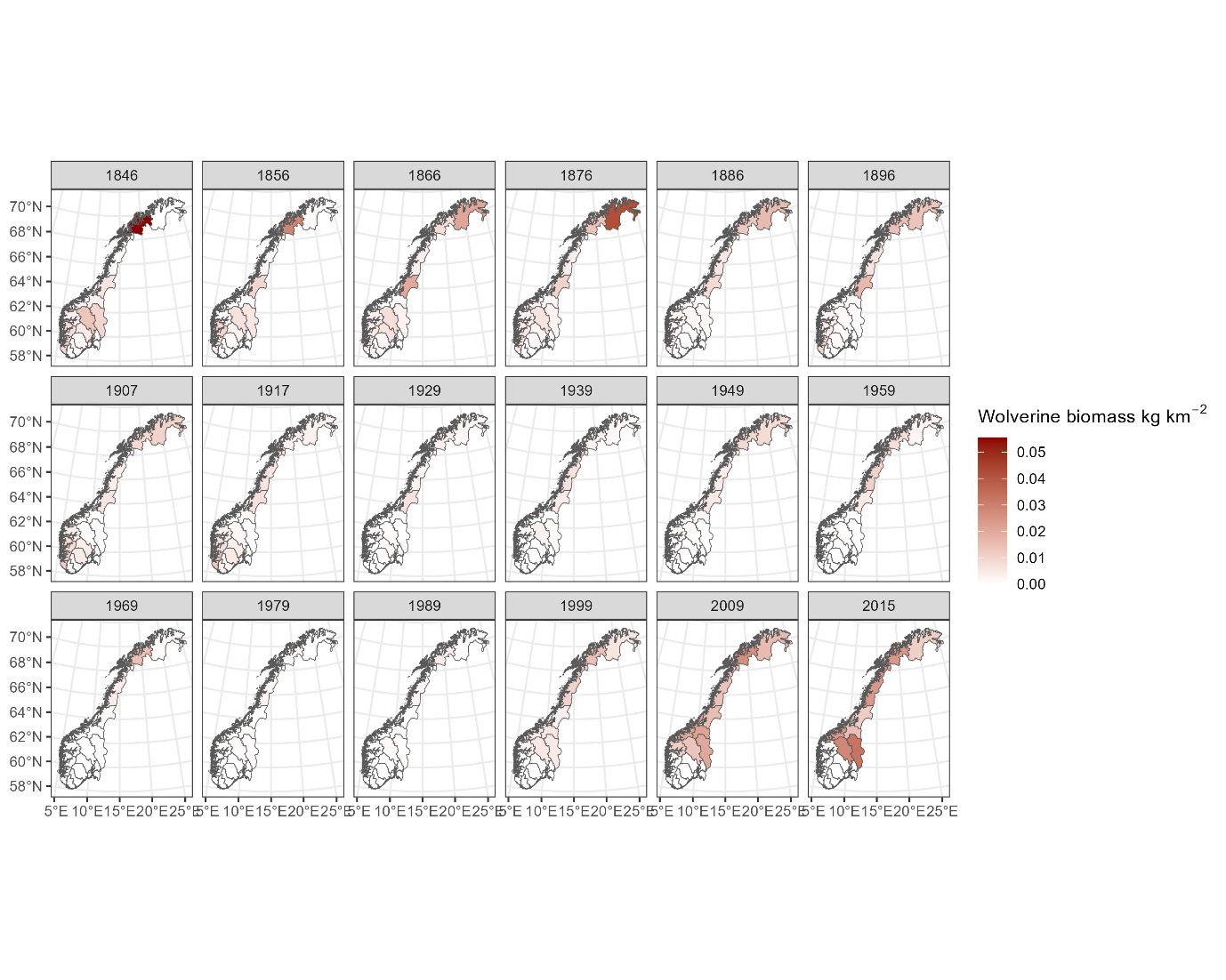


Figure S5d Wolverine biomass density (kg km^-2^) across Norway in selected years. Note that colour scales vary between species. The figures are suitable for temporal comparison within species but less suitable for comparison between species. Map created in R [22], using the package sf [24].


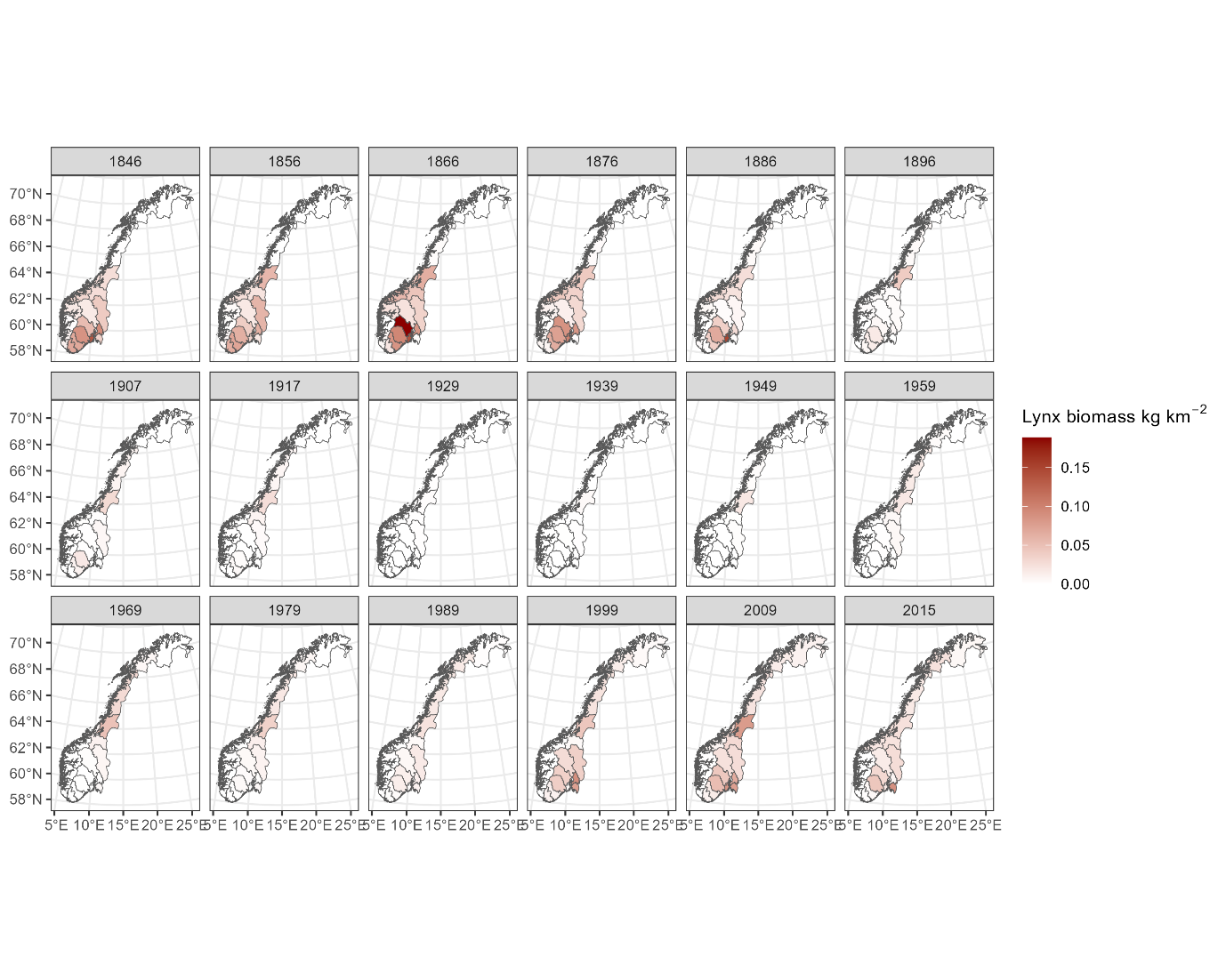


Figure S5e Lynx carnivore biomass density (kg km^-2^) across Norway in selected years. Note that colour scales vary between species. The figures are suitable for temporal comparison within species but less suitable for comparison between species. Map created in R [22], using the package sf [24].


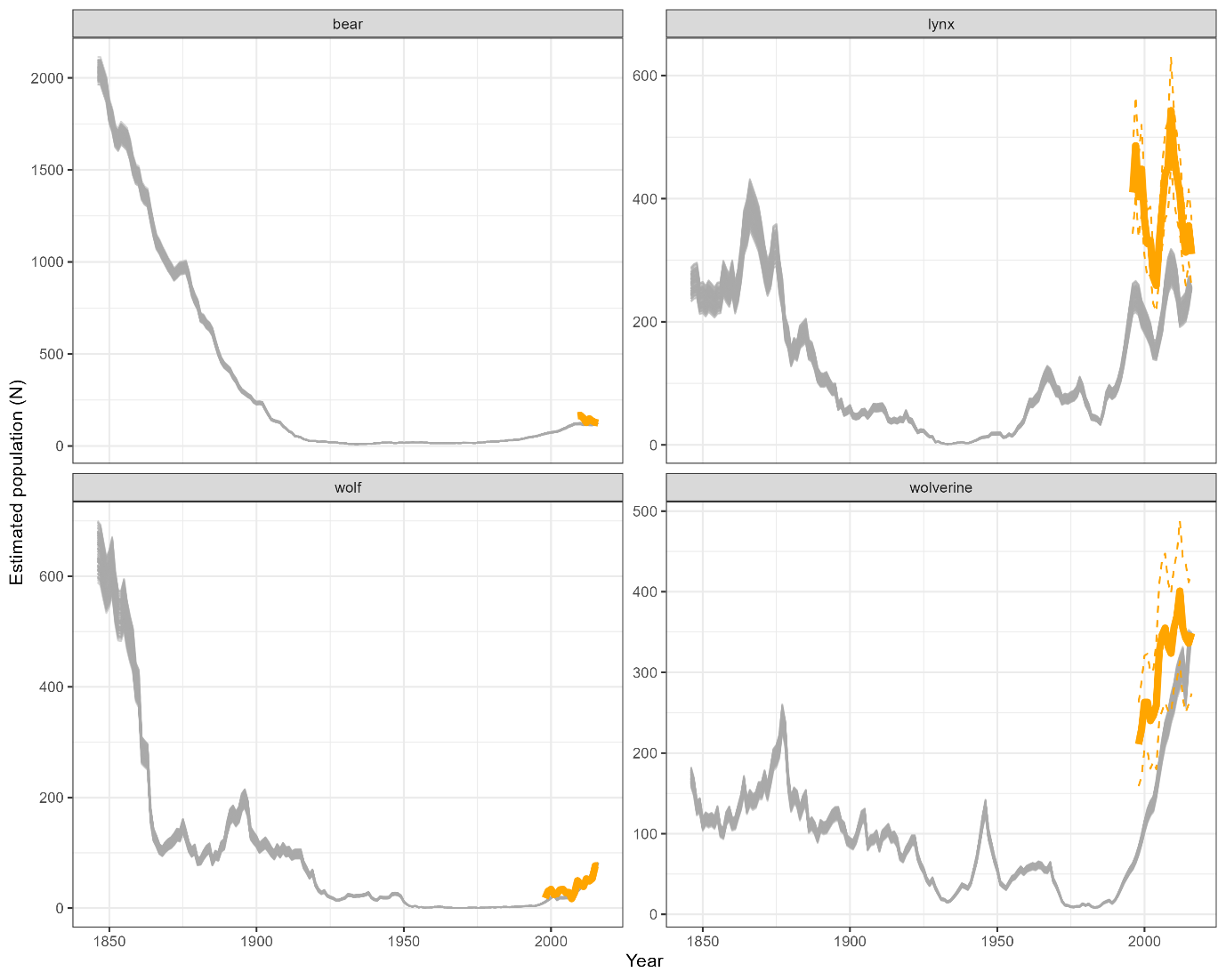


Figure S6 Backcast population trends for Norwegian carnivores from 1846 to 2016. Each line represents one of 100 simulations. Orange points show population sizes estimated through monitoring (RovData.no). Note that the backcast estimates are after hunting for a given year, while the monitoring estimates pertain to populations before the hunting season.
